# Potential probiotic and functional properties of *Brettanomyces* strains isolated from kombucha tea

**DOI:** 10.3389/fmicb.2024.1415616

**Published:** 2024-07-23

**Authors:** Lara Areal-Hermida, Pedro Coelho, Ángeles Pichardo-Gallardo, Cristina Prudêncio, Carmen Sieiro

**Affiliations:** ^1^Departamento de Bioloxía Funcional e Ciencias da Saúde, Área de Microbioloxía, Universidade de Vigo, Vigo, Spain; ^2^Centro de Investigação em Saúde Translacional e Biotecnologia Médica (TBIO)/Rede de Investigação em Saúde (RISE-Health), Escola Superior de Saúde, Instituto Politécnico do Porto, Porto, Portugal; ^3^ATC Ciências Químicas e das Biomoléculas, Escola Superior de Saúde, Instituto Politécnico do Porto, Porto, Portugal

**Keywords:** kombucha tea, probiotics, yeasts, *Brettanomyces*, antimicrobial activity, cytotoxicity, longevity

## Abstract

Kombucha, a beverage traditionally obtained through the fermentation of tea, is believed to have beneficial health properties. Therefore, characterizing the microorganisms responsible for this fermentation is essential to demonstrate its potential health benefits and to identify candidates for new probiotics. In this study, four probiotic yeast strains isolated from kombucha tea were identified, by the PCR-RFLP analysis of the ribosomal ITS region and the sequence of the D1/D2 domain of the 26S rDNA, as *Brettanomyces bruxellensis* (UVI55 and UVI56) and *B. anomalus* (UVI57 and UVI58). Properties relevant to probiotics were also studied in these strains. All of them showed excellent survival in simulated gastric (99%–100%) and duodenal (95%–100%) juices. The ability to self-aggregate (38%–100%), adhesion to xylene (15%–50%) and, above all, adhesion to Caco-2 cells (4%–21%), revealed its potential capacity to adhere to the intestinal epithelium. In addition, the tested strains showed excellent antioxidant capacity (82%–94%), antimicrobial activity against different pathogens (*Escherichia coli*, *Staphylococcus aureus*, *Salmonella enterica*, *Listeria monocytogenes*, and *Bacillus cereus*), as well as remarkable cytotoxic activity against colon, melanoma and ovarian tumor cell lines. Finally, using *Caenorhabditis elegans* as a model, strain UVI56 exhibited ability to both extend the lifespan of the nematode and protect it against infection by *S. enterica*. These results support the probiotic and functional properties of the analyzed strains. In conclusion, the study revealed that kombucha tea could be a source of potential probiotics that contribute to its health-promoting properties and that the characterized *Brettanomyces* strains could be exploited directly as probiotics or for the development of new functional foods.

## 1 Introduction

Probiotics are “live microorganisms that, when administered in adequate amounts, confer a health benefit on the host” (Food and Agriculture Organization of the United Nations and World Health Organization, 2006), with the remarkable ability to prevent and restore alterations in the microbiota (Latif et al., 2023). The gut microbiota is a complex ecosystem composed of bacteria, archaea, fungi and viruses that plays an essential role in human health (Lavelle and Hill, 2019). Alterations in the microbiota (dysbiosis) have been associated with numerous diseases (Lavelle and Hill, 2019). Modulation of the intestinal microbiota in order to maintain a favorable balance in the ecosystem and improve human health is therefore of great interest. In addition to the restoration of the intestinal microbiota, stimulation of the host’s immune system, activity against pathogens, antioxidant and anticancer properties, as well as the treatment of gastrointestinal disorders, among others, are health-promoting effects that probiotics can exert (O’Toole et al., 2017; Latif et al., 2023).

In general, most of the probiotic strains described and marketed belongs to Bifidobacteria and the group of lactic acid bacteria, often species of the genus *Lactobacillus* (Luang-In et al., 2020; Saadat et al., 2020). In contrast, *Saccharomyces cerevisiae* var. *boulardii* is the only yeast widely studied and marketed as a probiotic in both the pharmaceutical and food industries (Czerucka et al., 2007). Thus, *S. boulardii* is frequently used as a therapeutic agent to prevent or treat gastrointestinal disorders such as diarrhea, and its antimicrobial activity against *Clostridium difficile* has been demonstrated (Moré and Swidsinski, 2015; Goldenberg et al., 2017). In addition to *Saccharomyces boulardii*, other yeast species such as *Kluyveromyces marxianus*, *Torulaspora delbrueckii*, *Lachancea thermotolerans*, *Metschnikowia ziziphicola* and *Debaryomyces hansenii*, have also shown strain specific probiotic properties (Ochangco et al., 2016; Agarbati et al., 2020; Saadat et al., 2020; Hye-Youn et al., 2022). Some strains of these yeasts (mainly derived from fermented foods) have the ability to survive under digestive conditions and, among other properties, exhibit interesting antimicrobial activity against human pathogens (Agarbati et al., 2020). On the other hand, a distinguishing feature of yeasts is that they are naturally resistant to antibiotics, which makes them excellent candidates as new probiotics, very suitable for use in combination with antibacterial therapies (Neut et al., 2017).

The fermentation of different substrates to obtain new foods and beverages has been practiced since prehistoric times. These transformations, carried out by microorganisms (including yeasts), change the texture, nutritional composition and organoleptic properties of the product while often extending its shelf life. In addition, fermented foods are associated to beneficial health effects, mainly due to the microorganisms they contain and/or the compounds that these microorganisms can synthesize. Hence, the beneficial effects of microorganisms associated with fermented foods as promoters of human and animal health, are gaining more and more attention. Nevertheless, the confirmation of their probiotic potential and functional properties must be strictly demonstrated (Ilango and Antony, 2021). In addition, safety aspects must also be taken into account. In order to establish the safety of microorganisms used in food production, the European Food and Safety Authority (EFSA) proposed a safety assessment system for certain groups of microorganisms that establishes a qualified presumption of safety (QPS) status. Essentially, it was proposed that the safety assessment of a taxonomic unit could be based on four pillars: taxonomic identity, body of knowledge, potential pathogenicity and end use (EFSA-BIOHAZ Panel, 2007). Regarding food-derived probiotics, yeast strains with probiotic potential, as previously stated, have been isolated from some fermented foods, particularly from kefir, one of the most studied food sources (Gut et al., 2019; Hye-Youn et al., 2022). However, the yeasts of other fermented foods, in particular those of non-dairy origin, have been poorly studied. Kombucha is an acidic and slightly sweet drink, traditionally produced by the fermentation of sweet green or black tea (*Camelia sinensis*), through a symbiotic association of bacteria and yeasts that are organized into a cellulose biofilm known as SCOBY (Symbiotic Culture of Bacteria and Yeasts; Kapp and Sumner, 2019). The symbiotic association is composed of acetic bacteria, lactic acid bacteria and yeasts (Cardoso et al., 2020). Kombucha is believed to have originated from northeastern China (Manchuria) and is traditionally attributed with numerous health benefits that are still under investigation (de Miranda et al., 2022; Diez-Ozaeta and Astiazaran, 2022). Although different studies unveil great variability and diversity of the microbiota associated with kombucha, the *Acetobacteraceae* bacteria and the yeast families *Saccharomycetaceae* and *Schizosaccharomycetaceae* are predominant. Among the yeasts, the genus *Brettanomyces* appears as one of the most abundant (Villarreal-Soto et al., 2020; Pihurov et al., 2021).

Considering the importance of confirming the beneficial properties attributed to fermented foods, the growing interest in the probiotic potential of yeasts, and the demand for new probiotics with different characteristics, to be marketed as lyophilized cells or used for the development of new functional food products, the objective of this study was to evaluate the probiotic potential and possible functional properties of *Brettanomyces* strains isolated from kombucha tea that have not been studied so far.

## 2 Materials and methods

### 2.1 Isolation, inoculum preparation, and quantification of yeasts

Samples of artisanal kombucha (NW Spain, 43.361631, −8.411064) were diluted in saline solution [NaCl 0.85% (Panreac)], plated in WL nutrient agar (VWR Chemicals) medium and incubated at 30°C for 7 days in aerobic conditions. Four distinct morphotypes were differentiated and two colonies from each of the two most abundant morphotypes (Supplementary Figure S1) were re-isolated on Yeast extract-peptone-dextrose, YPD plates [2% Glucose (Scharlau), 2% peptone (Conda), 1% yeast extract (Labkem) and 1.5% agar (Labkem)] for purification and preserved in 20% glycerol at −80°C󠆳 for further work. To prepare a yeast inoculum, a yeast colony was transferred to a flask with 10 mL of YPD broth and incubated at 30°C for 48 h in aerobic conditions. This pre-inoculum was transferred to another flask with 100 mL of YPD broth and incubated at 30°C for 24 h. The cell density of this culture was estimated in a Neubauer’s chamber. Appropriate amounts of these cultures were centrifuged and the cells washed with saline solution to obtain the number of cells needed for each assay.

### 2.2 Yeasts identification and typing

Presumptive identification was carried out by the RFLP analysis of the 5.8S rRNA gene and the two ribosomal internal transcribed spacers (ITS region). The ITS regions were amplified with ITS1 (5′ TCC GTA GGT GAA CCT GCG G 3′) and ITS4 (5′ TCC TCC GCT TAT TGA TAT GC 3′) primers, and 10 μL of PCR products digested with HaeIII, HinfI and HhaII (CfoI) endonucleases and separated by electrophoresis on 4% agarose gels (Esteve-Zarzoso et al., 1999; Pham et al., 2011). Additionally, the D1/D2 domain, of the large ribosomal subunit (LSU) rRNA gene, was used to confirm yeasts identification (Kurtzman and Robnett, 1998). The D1/D2 region was amplified by PCR with NL1 (5′-GCATATATCAATAAGCGG AGGAAAAG-3′) and NL4 (5′-GGTCCGTGTTTCAAGACGG-3′) primers and sequenced. Identification was carried out by comparing the sequences obtained with those deposited in the databases using the NCBI Blast tool. Published sequences and sequences from strains belonging to culture collections and/or type strains were preferentially selected. Amplification of both, the D1/D2 and ITS regions, were carried out following the protocol of Esteve-Zarzoso et al. (1999) with modifications. The reactions, in a volume of 50 μL, consisted of 10 μM of dNTPs (Nzytech), 0.5 μM of each primer, 1.5 mM of MgCl_2_ (Nzytech), 10X Buffer and NZYProof DNA Polymerase (Nzytech) 2 U, using yeast cells as template. Amplification was performed in a thermocycler (MyCyclerTM Thermal Cycler System With Gradient Option) according to the following PCR conditions: initial denaturalization at 95°C during 2 min; 35 cycles of 30 s at 94°C, 30 s at 52°C, and 2 min at 72°C; and final extension at 72°C during 10 min. Since yeast cells were used as template, an extra pre-cycle at 95°C for 15 min was included. Yeast typing was carried out using the primers M13, M14, (GTG)_5_, (GAC)_5_, OPA02 and OPA09, following the protocol of Miot-Sertier and Lonvaud-Funel (2007). Yeasts DNA was extracted using the Microbial gDNA isolation Kit (Nzytech) following manufacturer instructions.

### 2.3 Preparation of cell free supernatants and intracellular extracts of yeasts

Cell free supernatants (CFS) were obtained by centrifugation of 48 h yeast cultures in YPD at 4,000 ×g for 5 min and filtered through 0.22 μm membrane filter and stored at −20°C (Sharma et al., 2020). The pellet obtained from the above centrifugation, was washed twice in Phosphate Buffer Saline, PBS (Sigma) and resuspended in the same volume of PBS. To obtain the intracellular extracts, glass beads were added (one third of the volume) and vortexing the mixture (10 min vortex/10 min ice-cold periods) until cell rupture. Finally, after centrifugation the supernatants were collected and stored at −20°C for further assays.

### 2.4 Bacteria cultures

For the antimicrobial activity assays the following bacterial strains were used: *Salmonella enterica* CECT 443, *Bacillus cereus* CECT 193, *Escherichia coli* CECT 433, *Staphylococcus aureus* CECT 231, *Listeria monocytogenes* CECT 5366. CECT: *Colección Española de Cultivos Tipo* (Spanish Type Culture Collection). For feeding nematodes a strain of *Escherichia coli* OP50 (Carolina Biological) was used. Strains were grown in Brain heart infusion, BHI (Cultimed) medium at 37°C overnight. When necessary, bacterial concentrations were adjusted to a turbidity equivalent to that of 0.5 MacFarland standard.

### 2.5 Cell lines and cell culture

Three different cell lines, Caco-2 (ATCC® HTB-37), Skov-3 (ATCC® HTB-77™) and B16F10 (ATCC® CCL-6475 ™), were used. All cells were grown in high-glucose Dulbecco’s modified Eagle’s medium (DMEM, Sigma-Aldrich) supplemented with penicillin and streptomycin 100 μg/mL (Gibco) and 10% fetal bovine serum (FBS, Hyclone). Cells were kept in a standard cell incubator at 37°C under a humid atmosphere with 5% CO_2_ (Menezes et al., 2020).

Complete medium was used for the growth and maintenance of cell lines and as internal positive control in the different assays. Serum-free DMEM, without FBS and supplemented with penicillin and streptomycin only, was employed as negative control in the different assays. All treatments with both yeast CFS and intracellular extracts were done in serum-free conditions.

### 2.6 *Caenorhabditis elegans* maintenance and synchronization

Nematode growth medium NGM [0.3% NaCl, 0.25% Peptone, 1.7% Agar, 0.1% cholesterol (5 mg/mL) (Sigma), 0.1% CaCl_2_ (1 M) (Panreac), 0.1% MgSO_4_ (1 M) (Panreac), 2.5% KH_2_PO_4_ (1 M) (Panreac) adjusted to pH = 6], and Nematode growth medium yeast extract NGMY (NGM + 4% yeast extract) were used for *Caenorhabditis elegans* growth and assays.

*Caenorhabditis elegans* N2 (wild-type) was acquired from Carolina Biological. The nematodes were routinely grown and maintained at 25°C on NGM plates, previously seeded with *E. coli* OP50 and incubated for 24 h at 37°C, according to Meneely et al. (2019).

The synchronization of *C. elegans* populations was also performed following the protocol described by Meneely et al. (2019), with minor modifications. In brief, to synchronize the population of *C. elegans* a bleach solution [48% of Bleach (5%) and 4% of NaOH (50%, w/w), Panreac] was used. After washing with M9 buffer [5.96% Na_2_HPO_4_ (Panreac), 2.99% KH_2_PO_4_, 1.28% NaCl, 0.25% MgSO_4_], the egg suspension was incubated overnight to obtain L1 larvae. Then, 200 μL of the L1 larval solution were transferred onto NGMY plates (previously seeded with *E. coli* OP50 as indicated above) which were incubated for 48 h at 25°C for the worms to reach the L4/young adult stage.

### 2.7 Evaluation of yeast hemolytic activity

The hemolysin production was evaluated following Gut et al. (2019) with modifications. Yeasts were inoculated by spots of 15 μL with 10^7^ cells on Columbia Blood Agar plates (Scharlau) in triplicate. Plates were incubated at 30°C and 37°C during 5 days and then inspected for alpha, beta or gamma hemolysis.

### 2.8 Yeast tolerance to temperature, low pH, and bile salts assay

Temperature and low pH survival assays were carried out according to Simões et al. (2021). Briefly, tubes containing 3 mL of YPD or 3 mL of YPD with the pH adjusted to 2 with an acidic solution (HCl 1 M), were inoculated with 10^8^ cells/mL and incubate at 37°C and 30°C, respectively, during 3 h. Bile salt tolerance experiments were carried as reported Basavaiah et al. (2019), with minor modifications. Briefly, tubes with 3 mL of YPD supplemented with 0.3% or 10% of Bile bovine Oxgall (Sigma Aldrich), were inoculated with 10^8^ cells/mL and incubated at 30°C for 4 h. Samples (0.1 mL) were taken at time 0 and after 3 h or 4 h (according to the assay), serially diluted, and plated on Malt Agar, MA [3% malt extract (VWH Chemicals) and 1.5% agar]. Plates were incubated at 30°C for 7 days and the viable cell counts (CFU/mL) determined. The survival rate was expressed by the population of viable cells considering the difference between initial and final time.

### 2.9 Resistance to simulated gastrointestinal conditions

The procedure was performed as described in Fadda et al. (2017), with minor modifications. First, 10^8^ cells/mL were inoculated in tubes with 5 mL of simulated gastric juice and after 90 min of incubation, cells were washed twice in saline solution and re-suspended in tubes with 3 mL of synthetic duodenum juice with 0.3% or 10% Bile bovine Oxgall. The survival rate was expressed by the population of viable cells (CFU/mL), considering the difference between initial and final time.

### 2.10 Determination of yeast aggregation

Yeast self-aggregation assay was performed according to Fadda et al. (2017). Briefly, an initial cell concentration of 10^7^ cells/mL in YPD was prepared. The cell suspension was maintained for 24 h at 37°C and optical density (OD) measurements at 600 nm taken after 2, 3, 4, and 24 h.

### 2.11 Yeast adhesion evaluation

Yeasts cell adhesion to hydrocarbons [chloroform (Labkem), ethyl acetate (Labkem) and xylene (PanReac)] was estimated by the MATH (Microbial Adhesion to Hydrocarbons) assay as described by Basavaiah et al. (2019). The yeast adhesion to the human colon adenocarcinoma cell line Caco-2 was carried out following Menezes et al. (2020).

### 2.12 Yeast antimicrobial activity

The ability of yeast strains to inhibit the growth of pathogenic bacteria was determined by the “spot on law” method described by Syal and Vohra (2013) with modifications. In this case, the plates with the yeast spots were previously incubated at 30°C for 24 h and the top layer prepared with 10 mL of TSB (0.7% agar) inoculated with 10^7^ cells/mL of the bacteria. To evaluate co-aggregation with different pathogenic bacteria, an initial concentration of 10^7^ cells/mL of both yeast and pathogens was used. The procedure was carried out as described by Ogunremi et al. (2015).

### 2.13 Antioxidant activity

Cells (10^7^ or 10^9^) harvested by centrifugation (12,000 rpm, 5 min) were washed twice with a sterile 0.9% NaCl solution. The resulting pellet was resuspended in 1 mL of the same solution and the evaluation of the antioxidant activity of the yeasts was performed by the DPPH (1,1-diphenyl-2-picrylhydrazyl) radical scavenging assay as previously described by Menezes et al. (2020).

### 2.14 Bile salt hydrolase activity

Bile salt hydrolase activity was estimated as described in Panicker et al. (2018) with minor modifications. Plates of bile salts were prepared by adding 0.5% (w/v) of bile salts (Sigma: Cholic acid sodium salt 50% and deoxycholic acid sodium salt 50%) and 0.37 g/L (w/v) of CaCl_2_ into YPD agar. These plates were inoculated with spots of 15 μL (with 10^7^ yeasts cells previously washed with PBS) and incubated at 30°C and 37°C aerobically during 6 days. The bile salts hydrolase activity was evaluated measuring the diameter of the precipitation zones around de colony: negative (no precipitation), low (<10 mm) medium (10–15 mm) and high (>15 mm; Gomes et al., 2008).

### 2.15 Cell cytotoxicity assay

The evaluation of the cytotoxicity of yeast CFS and intracellular extracts was carried out by the MTT assay using (3-(4,5-dimethylthiazol-2-yl)-2,5 diphenyl tetrazolium bromide) to determine the viable cells as described by Sharma et al. (2020) with modifications. Briefly, the assays were performed by seeding 100 μL of cell suspension (2 × 10^4^ cells/mL) in a 96/well cell culture plate. On the next day, cells were treated with 10, 20 and 40% of CFS or 10% of intracellular extracts in serum-free DMEM. DMEM with FBS and FBS-free DMEM were used as positive and negative controls, respectively. After 24 h of treatment, 10 μL of MTT (Sigma-Aldrich, Co.) reagent was added into each well, followed by a 3 h incubation period. Reduction of MTT to formazan in viable cells changed the yellow color of MTT to purple of formazan. Afterwards, 100 μL of dimethyl sulfoxide (Honeywell) was added into each well to dissolve the purple formazan precipitate. Absorbance was measured at 562 nm and 620 nm using an ELISA reader (EZ Read 800 Plus, Biochrom with Galapagos software 1.1.2.0, Biochrom). The results were calculated by the formula ABS (final) = ABS (562) − ABS (620) and normalized to control treatment (% of control). The assay was carried out in three independent experiments using triplicates.

### 2.16 *Caenorhabditis elegans* lifespan assay and infection preventive treatment

At the L4/young adult stage, the worms were divided into several groups. The control group was fed *ad libitum* on plates with *E. coli* OP50, and the experimental groups on plates with the yeast studied. The medium used was NGMY supplemented with 0.12 mM FudR (ThermoScientific). The plates were stored at 25°C until the death of the last worm. The number of live nematodes was scored daily (Poupet et al., 2019). To evaluate the preventive effects of yeasts against *S. enterica*, N2 nematode survival was measured in solid medium according to the work of Poupet et al. (2019), with modifications. L4/young adult stage nematodes were washed twice with M9 buffer and placed on NGMY +0.12 mM FudR plates, containing *E. coli* OP50 or yeast, at 25°C for 24 h. Then, nematodes were washed twice with M9 buffer, transferred onto BHI agar + 0.12 mM FudR plates (previously seeded with 10^8^ cells of *S. enterica* and incubated at 37°C during 24 h), and incubated at 25°C. The nematodes were observed daily and enumerated until the death of the last worm. The worms were considered dead when they did not move after mechanical simulation on the dish. The assays were carried out in three independent experiments with three Petri dishes (35 worms/plate) per condition.

### 2.17 Statistical analysis

Statistical analysis of data was carried out using GraphPad Prism Statistics software package version 9.4.0. One-way or two-way ANOVA followed by a Tukey’s posthoc was used to determine significant differences. Differences were considered statistically significant at *p* < 0.05. The *C. elegans* survival assay was examined using the Survival Analysis of Kaplan–Meier, and significant differences were determined using the Long-rank test.

## 3 Results

### 3.1 Identification and safety of the isolated yeasts

In the present study, from samples of artisanal kombucha grown in WL medium, after the analysis of 140 colonies, four distinct morphotypes were differentiated (Supplementary Figure S1). The most abundant morphotype accounted for 87% of the total colonies, followed by the other three morphotypes, which represented 10, 2 and 1% of the isolates, respectively. Two colonies from each of the two most abundant morphotypes were selected and identified. Isolates were identified by studying the ITS and D1/D2 regions of the rRNA. The profiles of the ITS RFLP analysis obtained for the four isolates allowed them to be assigned to two species of the genus *Brettanomyces* (Supplementary Table S1; Supplementary Figure S2). The sequencing of the D1/D2 rRNA region for the four isolates confirmed their assignment to the genus *Brettanomyces* and also established as the closest species *B. bruxellensis* for the isolates UVI55 and UVI56 (100% identity) and *B. anomalus* for the isolates UVI58 and UVI57 (99.67%–99.84% identity; Supplementary Table S1). Moreover, the D1/D2 sequences of the new isolates (Accession number OQ676194 for UVI55, OQ676217 for UVI56, OQ676309 for UVI57 and OQ676384 for UVI58) were submitted to GenBank. Additionally, random-amplified polymorphic DNA patterns generated with different markers were used for strain level differentiation. Thus, OPA02, (GTG)_5_, and M13 markers allowed isolates UVI57 and UVI58 to be established as different strains, while OPA02 and M14 markers allowed isolates UVI55 and UVI56 to be considered also as distinct strains (Supplementary Figure S3). Finally, no hemolytic activity (gamma hemolysis) was detected after incubation of the four isolates in blood agar for 5 days at 30°C and 37°C (Supplementary Figure S4).

### 3.2 Temperature and acid tolerance

For the four strains isolated in this study, stability at 37°C and in acid-rich medium (pH 2) was tested. With respect to temperature resistance (Table 1), all cells survived after 3 h exposure at 37°C. Regarding the tolerance to the acidic medium, all cells also survived for at least 3 h at pH 2 (Table 1). No differences between strains were observed in either case.

**Table 1:** Human temperature, acid and bile salts tolerance.

Yeast strains	Temperature (37°C)			pH 2			Bile salts					
							0.3%			10%		
	log CFU/mL			log CFU/mL			log CFU/mL			log CFU/mL		
	T0	T3	Survival (%)	T0	T3	Survival (%)	T0	T4	Survival (%)	T0	T4	Survival (%)
UVI55	7.814 ± 0.020	8.100 ± 0.115	103.660 ± 1.748^a^	7.788 ± 0.063	7.892 ± 0.003	101.336 ± 1.189^a^	8.035 ± 0.105	7.971 ± 0.020	99.213 ± 1.366^abA^	8.138 ± 0.030	8.033 ± 0.094	98.708 ± 0.943^aA^
UVI56	7.676 ± 0.135	7.984 ± 0.049	104.016 ± 2.141^a^	7.564 ± 0.069	7.731 ± 0.139	102.217 ± 2.333^a^	7.879 ± 0.065	7.695 ± 0.90	97.660 ± 1.772^aA^	8.121 ± 0.085	7.814 ± 0.091	96.222 ± 2.066^aA^
UVI57	8.471 ± 0.045	8.578 ± 0.088	101.257 ± 0.772^a^	8.368 ± 0.063	8.484 ± 0.100	101.386 ± 0.439^a^	8.131 ± 0.72	8.125 ± 0.033	99.680 ± 0.776^abA^	8.326 ± 0.046	8.125 ± 0.010	97.595 ± 0.606^aB^
UVI58	7.847 ± 0.122	7.916 ± 0.126	100.878 ± 2.136^a^	7.299 ± 0.065	7.553 ± 0.023	103.476 ± 0.893^a^	7.783 ± 0.056	7.975 ± 0.050	102.461 ± 1.339^bA^	7.818 ± 0.034	7.604 ± 0.133	97.268 ± 1.862^aB^

Values are given as mean ± SD of triplicates. Different lowercase letters indicate significant differences between the different strains (*p* < 0.05). Different capital letters indicate significant differences between different percentages of bile salts for the same yeast (*p* < 0.05).

### 3.3 Survival in simulated gastrointestinal conditions

Concerning survival in gastrointestinal conditions, the effect of bile salts on cell viability was first evaluated. Table 1 shows the results of the cell viability of the isolates tested after 4 h in the presence of two concentrations of bile salts: 0.3 and 10%. At a concentration of 0.3%, the survival of the strains ranged from 97% to 100%, with UVI55, UVI57 and UVI58 exhibiting the highest survival rates and UVI56 significantly lower survival. At a higher concentration of bile salts (10%), survival exceeds 96% in all studied strains, and no significant strain-to-strain differences were observed.

Afterwards, the survival of the isolates under simulated gastrointestinal conditions was assessed. Thus, cells were incubated in synthetic gastric juice (pH 2 and 0.3% pepsin) for 90 min and then exposed, for 180 min, in synthetic duodenal juice (pH 7.5, 0.1% pancreatin and 0.3 or 10% bile salts). The results are shown in Table 2. The survival rate after 90 min incubation in gastric juice was around 100% for all strains. After the additional incubation in duodenal juice with 0.3 or 10% bile salts, the survival of the cells ranged from 98% to 100% and 95% to 100%, respectively. For the concentration of 0.3% bile salts, strains UVI57 and UVI58 showed a slightly lower survival than that of UVI55 and UVI56. Increasing the concentration of bile salts to 10% equals all strains except UVI55 which stands out exhibiting the highest resistance.

**Table 2:** Survival in simulated gastrointestinal juices.

Strains	Bile salts (%)	Log CFU/mL _t0_	Log CFU/mL _t90_	Log CFU/mL _t180_	Survival _t90_ (%)	Survival _t180_ (%)
UVI55	0.3	7.904 ± 0.024	7.949 ± 0.068	7.978 ± 0.017	100.574 ± 0.669^aA^	100.942 ± 0.514^aA^
	10	8.092 ± 0.053	8.288 ± 0.361	8.334 ± 0.088	102.419 ± 4.055^aA^	102.992 ± 0.576^aA^
UVI56	0.3	7.744 ± 0.064	7.969 ± 0.065	7.863 ± 0.140	102.917 ± 1.573^aB^	101.537 ± 1.010^aA^
	10	8.071 ± 0.067	8.050 ± 0.055	7.875 ± 0.096	99,744 ± 0.414^aA^	97.570 ± 1.290^aB^
UVI57	0.3	8.102 ± 0.045	8.073 ± 0.039	7.961 ± 0.078	99,636 ± 0.643^aA^	98.266 ± 1.348^aB^
	10	7.817 ± 0.098	7.749 ± 0.023	7.500 ± 0.273	99.147 ± 1.538^aA^	95.972 ± 4.050^aB^
UVI58	0.3	7.976 ± 0.115	8.051 ± 0.030	7.603 ± 0.091	99.515 ± 1.111^aA^	98.495 ± 0.628^aAB^
	10	7.842 ± 0.049	7.804 ± 0.066	7.724 ± 0.061	100.953 ± 1.659^aA^	95.324 ± 0.293^bB^

Values are given as mean ± SD of triplicates. Different lowercase letters indicate significant differences (*p* < 0.05) in the survival in different juices (same concentration of bile salts) for the same strain. Different capital letters indicate significant differences (*p* < 0.05) between strains for each juice.

### 3.4 Adherence to intestinal cells

**Figure 1:**
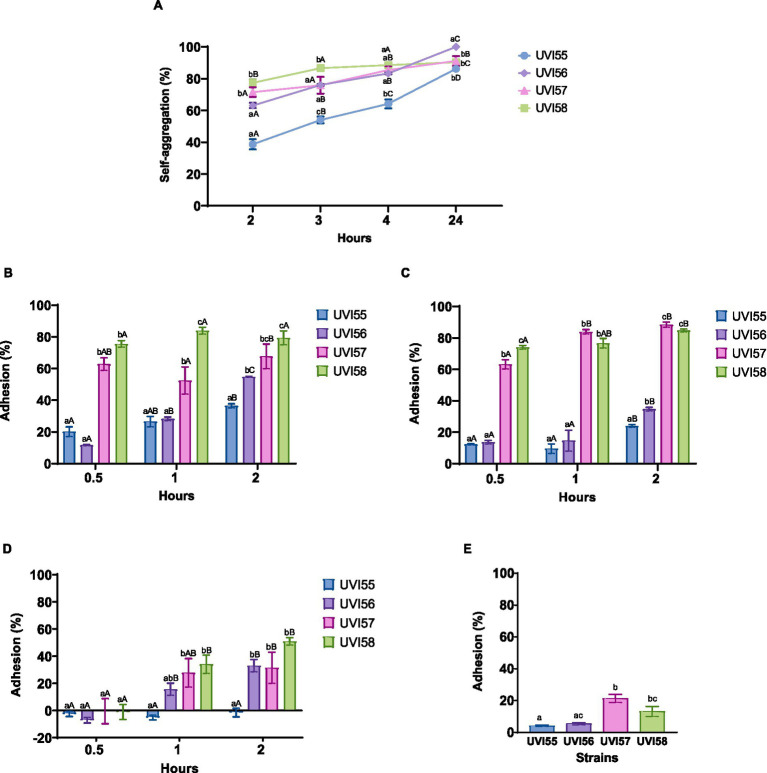
Percentage of yeasts self-aggregation **(A)**, adhesion to chloroform **(B)**, adhesion to ethyl acetate **(C)** adhesion to xylene **(D)** and adhesion to Caco-2 cells **(E)**. Values are given as mean ± SEM of triplicates **(A–D)** or three experiments in duplicate **(E)**. Different lowercase letters indicate significant differences (*p* < 0.05) between the different yeasts, and different capital letters indicate significant differences (*p* < 0.05) between different times of incubation for each yeast strain.

The ability of yeasts to adhere to intestinal cells was inferred by analyzing their self-aggregation and hydrophobicity and confirmed by testing their adherence to Caco-2 cells. The self-aggregation ability and cell surface properties, studied by the binding ability to different solvents, were first evaluated. Self-aggregating capacity (Figure 1A) increased over time (2–24 h) in all strains. After 2 h, the values ranged between 38% and 78%, highlighting the UVI57 and UVI58 strains with the highest values. After 4 h the self-aggregation values are equal for UVI56, UVI57 and UVI58 (around 88%), and different for UVI55 (64%). Finally, following 24 h of incubation the self-aggregation capacity was higher than 90% for all the strains, highlighting UVI56 that reaches 100%. The binding capacity to chloroform and ethyl acetate are shown in Figures 1B,C, respectively. The UVI55 strain showed the lowest adhesion capacity in both cases (around 36% for chloroform and 24% for ethyl acetate after 2 h) followed by the UVI56 strain, with UVI57 and especially UVI58 strains showing the most prominent adhesion capacity (around 75% for chloroform and 74% for ethyl acetate after 0.5 h). Figure 1D shows the binding capacity to xylene. Strain UVI55 did not show adherence to xylene at any of the times tested. With respect to the rest of the strains, UVI57 and UVI58 showed, after 1 h, a significantly higher percentage of adhesion to xylene than UVI56, the three strains being equal after 2 h (with a percentage of adhesion around 50%). Secondly, the ability of the strains to adhere to Caco-2 cells was evaluated. The results are presented in Figure 1E. All yeasts showed adherence to Caco-2 cells, ranging from approximately 4%–21%. Again, strain UVI55 showed the lowest percentage of adhesion, which increased significantly in the case of strains UVI57 and UVI58 (around 21%).

### 3.5 Bile salt hydrolase and antioxidant activities

The measured BSH activities are shown in Table 3 and Supplementary Figure S5. None of the studied strains showed BSH activity at 37°C. However, all strains, except *B. anomalus* UVI58, presented BSH activity on plates, after 48 h at 30°C (halos ranging approximately between 20 and 36 mm), with strain UVI57 exhibiting significantly higher bile salt deconjugation.

**Table 3:** Bile salt hydrolase activity.

Yeast strains	Halo (mm)		
	48 h 30°C	6 days 30°C	6 days 37°C
UVI55	20.000 ± 0.000^aA^	26.000 ± 5.657^aB^	-
UVI56	20.333 ± 0.577^aA^	24.667 ± 2.517^aA^	-
UVI57	36.000 ± 3.606^bA^	36.333 ± 3.215^bA^	-
UVI58	-	-	-

Values are given as mean ± SD of triplicates. Different lowercase letters indicate significant differences (*p* < 0.05) between yeasts at 48 h or 6 days of incubation and capital letters indicate significant differences (*p* < 0.05) between different incubation times for the same yeast.

**Figure 2:**
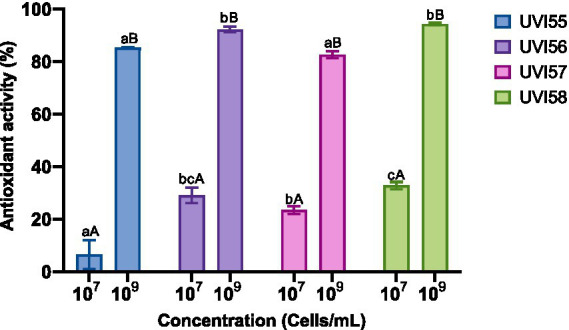
Antioxidant capacity of yeast strains. Values are mean ± SEM of triplicates. Different lowercase letters indicate significant differences (*p* < 0.05) for the same cell concentrations for different yeasts. Different capital letters indicate significant differences (*p* < 0.05) between different cell concentrations of the same yeast.

Afterwards, the antioxidant capacity of the *Brettanomyces* strains was assessed by determining DPPH scavenging activity using two different concentrations of cells (10^7^ and 10^9^). The results are shown in Figure 2. Antioxidant activity significantly increased as the number of yeast cells increased (from 6% to 32% with 10^7^ cells to 82%–94% with 10^9^ cells). Scavenging activities of around 80% were shown in other works (Cho et al., 2018) for 0.2 mM ascorbic acid. Strain UVI58 (10^7^ cells), as well as strains UVI56 and UVI58 (10^9^ cells) stand out with significantly scavenging activity.

### 3.6 Antimicrobial activity: antagonism and coaggregation with pathogens

The antimicrobial activity for the studied strains was first assessed throughout a microbial antagonism assay against five pathogens (*E. coli*, *S. enterica*, *S. aureus*, *B. cereus* and *L. monocytogenes*; Supplementary Figure S6) and the results are shown in Table 4. The strain *B. anomalus* UVI58 did not show antimicrobial activity against any of the pathogens tested under the assay conditions. The other strains showed antibacterial activity against some of the pathogens. Strain UVI57 was effective only against *Listeria monocytogenes* and *Bacillus cereus*, showing the same effectiveness against both strains. In contrast, strains UVI55 and UVI56 were active against all the pathogens tested and no significant differences, in the degree of efficacy, were observed either between them or against the different pathogens.

**Table 4:** Antimicrobial activity of yeasts strains against different pathogens.

Yeast strains			Halo (mm)		
	*Escherichia coli*	*Staphylococcus aureus*	*Salmonella enterica*	*Bacillus cereus*	*Listeria monocytogenes*
UVI55	14.667 ± 1,528^aA^	14.844 ± 2.566^aA^	14.667 ± 2.517^aA^	16.333 ± 2.309^aA^	14.333 ± 1.155^aA^
UVI56	19.667 ± 9.074^aA^	15.333 ± 3.512^aA^	14.667 ± 4.619^aA^	16.00 ± 5.000^aA^	15.667 ± 6.429^aA^
UVI57	-	-	-	15.833 ± 4.252^aA^	13.667 ± 1.528^aA^
UVI58	-	-	-	-	-

Values are given as mean ± SD of triplicates. Different lowercase letters indicate significant differences (*p* < 0.05) in the efficacy of the same yeast against different pathogens. Different capital letters indicate significant differences (*p* < 0.05) between yeast strains for the same pathogen.

The coaggregation capacity was also tested in the strains characterized in this study, at different time points and for four pathogens (*E. coli*, *S. enterica*, *S. aureus*, *B. cereus*). The results are presented in Table 5. All the examined yeasts showed coaggregation capacity with all the pathogens tested. Coaggregation was observed between 2 and 4 h, varying according to the yeast strain and pathogen, and increased significantly, as the incubation time increased in most of the assays. No significant differences were observed between strains after 24 h. It is remarkable that the coaggregation percentages exceed 55% after 4 h for more than half of the studied combinations. Thus, the percentage of coaggregation of UVI55 and UVI57 with *E. coli*, that of UVI57 and UVI58 with *S. enterica* and *S. aureus*, and that of UVI55, UVI57 and UVI58 with *B. cereus* after 4 h, stands out significantly.

**Table 5:** Coaggregation of yeasts strains with pathogens.

Yeast strains	2 h	4 h	24 h
***Escherichia coli***			
UVI55	47.103 ± 10.725^aA^	55.270 ± 14.106^abcA^	85.212 ± 1.895^aB^
UVI56	9.905 ± 4.091^bA^	46.770 ± 5.778^bB^	87.666 ± 0.523^aC^
UVI57	48.998 ± 4.574^aA^	68.080 ± 2.949^cB^	82.623 ± 1.986^aC^
UVI58	82.709 ± 1.612^cA^	52.538 ± 2.659^abcB^	85.171 ± 3.644^aA^
***Salmonella enterica***			
UVI55	16.742 ± 7.309^aA^	42.457 ± 17.866^aB^	91.317 ± 0.321^aC^
UVI56	−4.415 ± 1.583^bA^	30.615 ± 1.817^aB^	88.192 ± 1.479^aC^
UVI57	49.262 ± 4.299^cA^	68.935 ± 3.489^bB^	94.503 ± 1.868^aC^
UVI58	63.010 ± 9.997^cA^	81.556 ± 4.655^bB^	87.359 ± 0.226^aB^
***Staphylococcus aureus***			
UVI55	5.374 ± 7.888^aA^	33.105 ± 3.958^aB^	82.762 ± 1.998^aC^
UVI56	2.556 ± 4.038^aA^	46.525 ± 8.663^bB^	85.655 ± 1.705^aC^
UVI57	66.640 ± 5.001^bA^	84.104 ± 5.225^cB^	93.085 ± 1.799^aB^
UVI58	76.253 ± 1.274^bA^	72.822 ± 2.545^cA^	87.688 ± 1.471^aB^
***Bacillus cereus***			
UVI55	25.885 ± 8.322^aA^	72.172 ± 3.228^aB^	86.330 ± 5.780^aC^
UVI56	9.578 ± 9.245^bA^	24.456 ± 3.002^bB^	88.980 ± 2.542^aC^
UVI57	45.727 ± 3.451^cA^	66.969 ± 0.547^aB^	89.711 ± 1.125^aC^
UVI58	55.697 ± 3.335^cA^	74.085 ± 2.041^aB^	81.624 ± 2.162^aB^

Values are given as mean ± SD of triplicates. Different lowercase letters indicate significant differences (*p* < 0.05) between the different strains for each incubation time. Different capital letters indicate significant differences (*p* < 0.05) between the different incubation times for each of the yeast strains.

### 3.7 Cytotoxic activity of yeast supernatants and yeasts extracts

**Figure 3:**
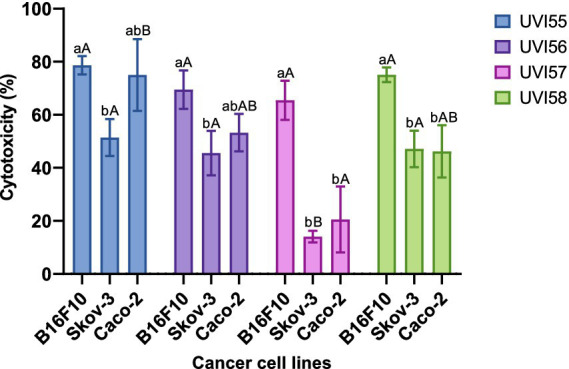
Percentage of cytotoxicity of 10% intracellular extracts of different yeasts against B16, Skov-3 and Caco-2 cell lines normalized with the negative control (PBS). Values are mean ± SEM of three experiments in triplicate. Different lowercase letters indicate significant differences (*p* < 0.05) between the efficacies against different tumor cell lines for the same yeast. Different capital letters indicate significant differences between different yeasts for the same tumor cell line.

Cytotoxic activity was evaluated, for the yeast strains examined in this work, against the malignant cell lines Caco-2 of colorectal cancer, B16F10 of melanoma and Skov-3 of ovarian cancer. For this purpose, the yeasts were cultured in YPD medium in order to guarantee their optimal growth. Then, the supernatants and the cells from which the intracellular extracts were obtained were processed separately. For the supernatants it was not possible to detect cytotoxic activity when compared with YPD culture medium itself (data no shown). In contrast, the cell extracts of all strains (used at 10%) showed a very remarkable cytotoxic activity against all cell lines tested (Figure 3) ranging between 14% and 78% depending on the strain and the tumor cell line. The highest degree of cytotoxicity was observed against the B16F10 cell line (around 78%), with all the yeast strains showing similar cytotoxic activity. In the case of the other cell lines, except for strain UVI57, which showed a cytotoxicity of around 14% against Skov-2 and 20% against Caco-2, the other yeasts showed the same effectiveness (around 50%).

### 3.8 Effects of *Brettanomyces bruxellensis* UVI56 on *Caenorhabditis elegans* survival and prevention of *Salmonella enterica* infection

**Figure 4:**
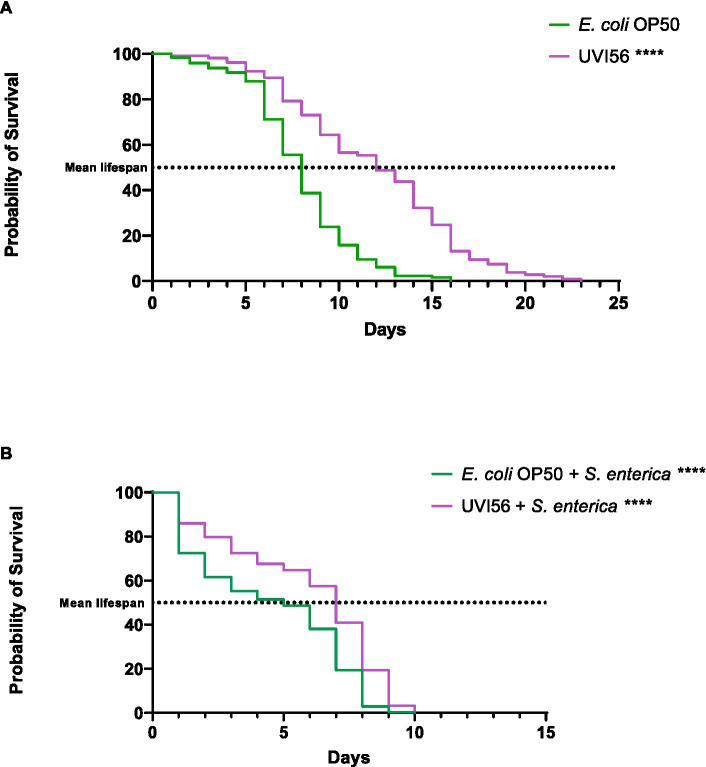
Influence of *Brettanomyces bruxellensis* UVI56 and *Escherichia coli* OP50 on the lifespan of *Caenorhabditis elegans* wiltd-type N2 strain **(A)** and preventive effect of *B. bruxellensis* UVI56 exposure on survival of *C. elegans* infected with *Salmonella enterica* **(B)**. Values are means of three assays in triplicate. Mean lifespan, with half of the population dead, is represented on the abscissa. The asterisks indicate the *p*-values (long-rank test) with *E. coli* OP50 as a control (^****^p < 0.0001).

In this work, strain UVI56 showed excellent probiotic properties and antimicrobial activity against all the pathogens tested. Therefore, it was selected to study its effect on the longevity of *C. elegans* and its ability to protect the worm against infection by *S. enterica*, a foodborne pathogen that causes one of the most common intestinal infections. Thus, the effect of *B. bruxellensis* UVI56 strain feeding on the lifespan of *C. elegans* N2 instead of the *E. coli* OP50 strain (conventional food of the nematode), was evaluated. The results are shown in Figure 4A. First, *B. bruxellensis* increased (significant *p* < 0.0001) the mean lifespan of worms by 4 days compared to the worms fed with *E. coli* OP50 (12 vs. 8 days). Next, the potential capacity of the yeast UVI56 to prevent the effects of infection of *C. elegans* by *S. enterica* was explored. For this purpose, the nematodes were first fed with *E. coli* OP50 or *B. bruxellensis* UVI56 and subsequently infected with the pathogen. The results are presented in Figure 4B. It was observed that worms, both those fed with the yeast and their usual food had their longevity reduced, between 6 and 12 days, compared to uninfected nematodes. However, nematodes fed with UVI56 and infected with *S. enterica* showed a significantly (*p* < 0.0001) longer mean lifespan compared to nematodes previously fed with *E. coli* (7 vs. 5 days).

## 4 Discussion

Potential probiotic microorganisms have been successfully isolated from various fermented foods and drinks (Gut et al., 2019; Hye-Youn et al., 2022; Ntiantiasi and Lianou, 2023). Thus, fermented foods represent a large pool of microorganisms and suitable candidates for the discovery of novel yeast strains with probiotic effects and functional properties. The study of the microorganisms involved in fermented foods is also essential to demonstrate their potential beneficial properties for health. However, the selection of probiotic strains should first consider safety aspects including the origin of the strain, its identification and absence of harmful activities (Food and Agriculture Organization of the United Nations and World Health Organization, 2006).

In the present study the WL medium, previously used in numerous studies to differentiate wine yeasts (Ghosh et al., 2015), was also suitable for the differentiation of yeasts isolated from kombucha tea. The ITS RFLP analysis (Esteve-Zarzoso et al., 1999) and the sequence of the D1/D2 region of the rRNA (Kurtzman and Robnett, 1998), previously used successfully in the identification of yeasts, allowed the identification of four colonies belonging to the most abundant morphotype in WL as *B. bruxellensis* (UVI55 and UVI56) and *B. anomalus* (UVI57 and UVI58). Additionally, random-amplified polymorphic DNA patterns generated with different markers (Miot-Sertier and Lonvaud-Funel, 2007; Crauwels et al., 2014) confirmed the differentiation of the four isolates at the strain level. Considering that the strains are originated from a beverage for human consumption and that the non-hemolytic strains are good candidates in terms of food safety, they were considered probably safe. However, their safety needs to be confirmed by taking into account the requirements they should meet to be considered QPS microorganisms and ensuring safety at the strain level (Miguel et al., 2022).

From ingestion until they reach the intestine, probiotics must remain viable to exert their beneficial effects. In order to do so, probiotic microorganisms must resist the human body temperature, particularly when they are not of human origin, as well as the adverse and aggressive conditions of the stomach (presence of acid and pepsin; Food and Agriculture Organization of the United Nations and World Health Organization, 2006; Binda et al., 2020; Han et al., 2021).

Practically all of the cells of the four strains studied in this work survived at body temperature for 3 h. The same survival rate was observed at pH 2 over the same period, which was not surprising given the origin of the strain. Resistance to acidic environments, individually evaluated in other yeast species, under the same conditions, varied between 56% and 100% (Syal and Vohra, 2013; Ogunremi et al., 2015; Simões et al., 2021; Lata et al., 2022), placing the kombucha-isolated yeasts, analyzed in this study, at the highest levels of acid-resistance. The resistance to the acidic medium has been attributed to the adaptation of cell walls and to the general routes of stress response (Kapteyn et al., 2001; Lucena et al., 2020).

After reaching the intestine, probiotic cells should be able to withstand prolonged exposures to bile salts, digestive enzymes such as pancreatin, and alkaline stress. In addition, probiotics must be able to remain in the intestinal tract as long as possible, otherwise their beneficial effects would be limited (Food and Agriculture Organization of the United Nations and World Health Organization, 2006; Binda et al., 2020; Han et al., 2021).

Tolerance to a concentration of at least 0.3% of bile salts is critical for the selection of probiotic microorganisms (Ogunremi et al., 2015), so resistance to bile salts was evaluated first. The four strains tested, with minimal variability, showed excellent tolerance to a bile salt concentration of 0.3%. These results are in accordance with data unveiled by other authors for other yeast genera and species (Syal and Vohra, 2013; Ogunremi et al., 2015; Simões et al., 2021). Additionally, a bile salt concentration of 10% showed minimal impact on cell survival.

The analysis of the yeasts under study in simulated gastrointestinal conditions, which allowed the simultaneous evaluation of the resistance of the yeasts to the major set of adverse factors they must face along the digestive tract, showed a very high resistance of the strains. The viability was not affected in gastric juice and minimally after passage through duodenal juice (both with 0.3 and 10% bile salts), with a survival of 95% or more under the most severe conditions, exhibiting *B. bruxellensis* strain UVI55 the highest resistance. For bile salt concentrations between 0.1–0.3%, numerous studies with other yeast strains, although in assays conducted under distinct conditions, have reported survival rates between 1.6% and 93% under synthetic gastric and duodenal juice incubation, and between 81% and 100% for exposure to 10% bile salts (Ogunremi et al., 2015; Fadda et al., 2017; Andrade et al., 2019; Goktas et al., 2021). According to Pennacchia et al. (2008), survival in gastrointestinal conditions must be higher than 70% to consider a microorganism as having probiotic potential. The results obtained for the *Brettanomyces* strains analyzed in this work are equal or exceed those obtained for other yeasts in the most drastic conditions (Ogunremi et al., 2015; Andrade et al., 2019; Goktas et al., 2021), so they can be considered to have a high probiotic potential in terms of their survival in the gastrointestinal tract.

The ability to colonize the gut epithelium, at least transiently, is essential for a probiotic to avoid rapid elimination, as well as to compete with pathogens for colonization of the gut. The adhesion of probiotics to the gut epithelium is a complex process involving specific and non-specific mechanisms (Sourabh et al., 2011). The adhesion capacity has been assessed for the yeasts isolated in this work, first indirectly, by studying their cell surface properties, that is, through the capacity for self-aggregation and hydrophobicity of their cell surface. The self-aggregation capacity ensures that the probiotic reaches a high cell density in the gut contributing to the adhesion mechanisms, and a strong hydrophobicity of the cell surface allows an improved interaction between the microorganism and the epithelial cells (de Melo Pereira et al., 2018). The self-aggregation of the four strains tested increased over the studied time (24 h), highlighting, after 2 h, the strains of *B. anomalus* UVI57 and UVI58 with the highest values (around 71%), which stand out when compared to those found for other species (Fadda et al., 2017; Amorim et al., 2018; Andrade et al., 2019; Gut et al., 2019). After maximum incubation time, self-aggregation rates reach 90% or 100% (for *B. bruxelensis* UVI56). These results are in agreement with those found for other yeasts (Syal and Vohra, 2014; Ogunremi et al., 2015; Amorim et al., 2018; Sakandar et al., 2018; Di Cagno et al., 2020; Goktas et al., 2021; Hye-Youn et al., 2022; Ntiantiasi and Lianou, 2023). The cell surface properties of the studied strains were examined by their ability to adhere to the solvents chloroform (as polar acidic solvent), ethyl acetate (as polar basic solvent) and xylene (as apolar solvent). The electron donor (basic) and electron acceptor (acidic) characteristics of bacterial surface were established by their ability to bind to chloroform and ethyl acetate, respectively. The results showed that the adhesion to both solvents vary with the strain showing *B. bruxellensis* UVI55 the lowest adhesion capacity and highlighting *B. anomalus* UVI57 and particularly UVI58 with higher values (around 74% after 0.5 h) than those found by other authors (Helmy et al., 2019). Hydrophobicity was evaluated against xylene. Except for *B. bruxellensis* UVI55, all strains showed adherence to xylene showing differences after 1 h (highlighting *B. anomalus* UVI57 and UVI58 vs. *B. bruxellensis* UVI56) and matching with a percentage of adhesion of around 50% after 2 h. These results are among those found for species of other yeast genera, although these studies show, in different cases, higher values against xylene at shorter times (Amorim et al., 2018; Sakandar et al., 2018; Basavaiah et al., 2019; Di Cagno et al., 2020). Considering the results of self-aggregation and hydrophobicity together, the analyzed strains would have a significant colonization capacity as the microbial adhesion to gut epithelium is more related to self-aggregation than to hydrophobicity (Del Re et al., 2000).

Human colon tumor cell lines, such as Caco-2, are considered a good model to assess the ability of microorganisms to adhere to the intestinal mucosa (Cho et al., 2018; Menezes et al., 2020). To gain further insight into their colonization capacity, adherence to Caco-2 was evaluated for the strains characterized in this study. All yeasts tested showed ability to adhere to Caco-2 cells, which varies depending on the strain. Again, and in agreement with the previously presented results regarding self-aggregation and hydrophobicity, the lowest percentage of adherence was shown by the *B. bruxellensis* UVI55 strain, highlighting with significantly higher percentages the *B. anomalus* UVI57 and UVI58 strains (about 21%). The adherence levels observed are in line with those already reported in the literature for other strains (Fadda et al., 2017).

Apart from surviving along the human gut, probiotics must also demonstrate properties directly beneficial to health. Therefore, for the strains characterized in this study different biological and functional properties were also evaluated.

BSH is one of the mechanisms by which probiotic strains can reduce serum cholesterol levels (Liong and Shah, 2005). All strains studied in this work, except *B. anomalus* UVI58, presented BSH activity at 30°C, highlighting *B. anomalus* UVI57, although none showed activity at 37°C. For other yeasts tested in earlier studies as probiotics, BSH is also strain variable (Fadda et al., 2017; Fernández-Pacheco et al., 2021). This bile salt deconjugation capacity could increase the interest of the positive strains of this study, although only to a limited extent considering that it was not detected at 37°C. Nevertheless, it is not yet completely understood whether high levels of BSH are in fact beneficial since large amounts of deconjugated bile salts could have undesirable effects in humans (Begley et al., 2006). BSH activity has also been considered relevant for the survival of probiotic microorganisms in the presence of bile salts in the duodenum (Pisano et al., 2014; Owusu-Kwarteng et al., 2015; Panicker et al., 2018). Contrarily, several authors have reported that resistance to bile salts toxicity in yeasts is independent of BSH activity (Sourabh et al., 2011; Şanlidere Aloğlu et al., 2016) and other mechanisms may be involved (Noriega et al., 2006). In fact, the strain UVI58 presents high resistance to bile salts despite no detectable BSH activity was observed.

Regarding the antioxidant capacity demonstrated for the *Brettanomyces* strains analyzed using 10^7^ cells, it is in accordance (in the case of UVI56, UVI57 and UVI58) with what was reported by Cho et al. (2018). This antioxidant power was shown to be dependent on the number of cells increasing significantly by increasing the number of cells to 10^9^. Gil-Rodríguez et al. (2015) established five groups of yeasts according to the percentage of antioxidant activity: very low (<20%), good (30–40%), very good (40–50%) and excellent (>50%). Accordingly, the kombucha-isolated strains, included in this study (with an antioxidant activity between 82–94%), would be classified as holding excellent antioxidant capacity. It is believed that the antioxidant activity of yeasts is mainly due to the content of (1,3), (1,6)-beta-D-glucan and protein fractions found in cell walls (Jaehrig et al., 2007).

One of the most desirable properties of probiotics is their antimicrobial activity against pathogens (Amorim et al., 2018). In the case of the strains characterized in this study, except for *B. anomalus* UVI58, all showed antimicrobial activity against important pathogens that may cause intestinal infections (*E. coli, S. enterica, S. aureus, B. cereus* and *L. monocytogenes*), with *B. bruxellensis* UVI55 and UVI56 showing a general efficacy against all strains tested and *B. anomalus* UVI57 a selective action only against the Gram-positive *Listeria monocytogenes* and *Bacillus cereus*. In previous studies, Agarbati et al. (2020) also demonstrated antibacterial activity in *Brettanomyces* strains isolated from beer. Antibacterial activity is infrequent in the probiotic yeasts studied so far and is, in fact, very variable depending on the strain (Fadda et al., 2017; Andrade et al., 2019; Basavaiah et al., 2019; Lata et al., 2022). Antibacterial activity is associated with the production of antagonistic molecules such as peptides, organic acids, proteases or polyamines (Zaouche et al., 2000; Murzyn et al., 2010; Naimah et al., 2018). In this sense, most of the isolates characterized in this study exhibited remarkable antibacterial properties.

Coaggregation is another interesting property of probiotics that mechanically prevents colonization of the intestine by pathogens (Ogunremi et al., 2015; Simões et al., 2021). All the strains tested stood out for their ability to coaggregate (exceeding 55% after 4 h for most of the combinations) with the four pathogens included in the study (*E. coli*, *S. enterica*, *S. aureus, B. cereus*). Much lower percentages, ranging from 0–55% after 4 h, have been described for other yeasts with different pathogens (Ogunremi et al., 2015; Simões et al., 2021). It has been proposed that this coaggregation capacity is based on the fact that certain pathogenic bacteria have on their surface binding molecules that allow them to adhere to yeasts via mannane and other polysaccharides on their cell wall (Ogunremi et al., 2015). Therefore, the new strains evaluated herein disclose a remarkable antibacterial activity which is of great interest, either at the intestinal level to compete with pathogens or to be used in future functional foods where they could additionally act as preservatives.

Probiotics, paraprobiotics and postbiotics, particularly those of bacterial origin, have been proposed for the treatment and prevention of cancer (Kumar et al., 2010; Delesa, 2017; Chuah et al., 2019; Luang-In et al., 2020; Latif et al., 2023). Several mechanisms have been proposed as responsible for this anticancer activity, including different bioactive cytotoxic metabolites such as short chain fatty acids or exopolysaccharides (Delesa, 2017; Luang-In et al., 2020). The cell extracts of the strains tested showed very remarkable cytotoxic activity against all or some of the cell lines tested, varying according to the strain-cell line combination. The activity of the four strains against the malignant cell line B16F10, as well as that of *B. bruxellensis* UVI55 and UVI56 and *B. anomalus* UVI58 against Caco-2 of colon cancer and Skov-3 of ovarian cancer is noteworthy. When compared with that of bacteria (Delesa, 2017), the cytotoxic activity of probiotic yeasts is scarcely studied, although different authors have also demonstrated cytotoxic activity for other yeast species (Luang-In et al., 2020; Ragavan and Das, 2020; Pakbin et al., 2022) against different cell lines. However, as far as we know, this is the first time that the cytotoxic activity of yeasts against B16F10 and Skov-3 cell lines has been demonstrated.

*Caenorhabditis elegans* is considered a potent *in vivo* model for carrying out prolongevity studies (Roselli et al., 2019), as well as for studying the pathogenicity of microorganisms and the antimicrobial properties of probiotics (Poupet et al., 2019). Given the previous results obtained, particularly the antibacterial properties previously analyzed, strain *B. bruxellensis* UVI56 was chosen to evaluate its effect on longevity using this model. The results demonstrated that the UVI56 strain increases the mean lifespan of the worms compared to that of worms fed with their usual food. Different probiotic strains have been shown to exert anti-aging effects on nematodes by acting on common molecular pathways such as insulin/insulin-like growth factor-1 (IIS) and p38 mitogen-activated protein kinase (p38 MAPK; Poupet et al., 2019; Roselli et al., 2019). Although very few studies explore the effect of probiotic yeasts on *C. elegans*, Veisseire et al. (2020) in solid medium also demonstrated an increase of 1 and 4 days in the mean lifespan of the nematode with *Saccharomyces cerevisiae* strain Sc16 and *Debaryomyces hansenii* strain Dh25, respectively. However, it is important to note that this effect is strain-dependent since the same authors showed that *S. boulardii* strain Sb1079 decreased the mean lifespan of worms by 2 days. It was also proved that infection with *S. enterica* reduces the longevity of nematodes, however, under these conditions, the UVI56 strain also increased the mean lifespan of worms when compared to those ingesting the conventional food, demonstrating a protective effect of the yeast against infection. Other studies have also unveiled a protective effect of other probiotic yeasts against other pathogens (Kunyeit et al., 2019; Veisseire et al., 2020). Thus, it was shown that *B. bruxellensis* (strain UVI56) increases *C. elegans* lifespan, and this rise in mean lifespan rates is maintained even after infection with a pathogen.

## 5 Conclusion

In conclusion, the results presented show that the studied strains of *Brettanomyces* species, isolated from kombucha tea, demonstrate*, in vitro*, a great resistance to human body temperature and adverse gastrointestinal conditions, as well as a great ability to adhere to the intestinal epithelium, with inherent strain variability. Additionally, these kombucha-isolated yeasts presented excellent functional properties, including both antioxidant and antimicrobial activity against various pathogens (except UVI58 strain) and cytotoxic effects against colon, melanoma and ovarian tumor cell lines. Additionally, the UVI56 strain enhanced the lifespan of *C. elegans*, while providing a protective effect of the nematode against infection by *S. enterica*. Accordingly, the *B. bruxellensis* and *B. anomalus* strains characterized in this study have a remarkable probiotic potential, at least equivalent and sometimes even superior to that of other previously characterized yeast strains.

Although additional *in vivo* assays are of paramount importance to further elucidate the biological mechanisms involved and confirm the goodness of these yeasts, the current study demonstrates that these strains of *Brettanomyces*, in many cases the dominant species in the consortium of microorganisms used for the production of kombucha tea, hold numerous key biological activities and therapeutic properties, thus synergically supporting the beneficial effects and probiotic potential that kombucha tea beverages entail.

Altogether, these strains of *Brettanomyces* could also be exploited directly as probiotics, representing a novel product for the probiotics market, or be used in the development of new functional foods. New value-added food products, particularly of plant origin, or traditional fermented beverages could be manufactured from these probiotic yeasts by food processing industries.

## Data availability statement

The datasets presented in this study can be found in online repositories. The names of the repository/repositories and accession number(s) can be found at: https://www.ncbi.nlm.nih.gov/genbank/, OQ676194; OQ676217; OQ676309; and OQ676384.

## Ethics statement

The manuscript presents research on animals that do not require ethical approval for their study.

## Author contributions

LA-H: Formal analysis, Methodology, Validation, Writing – review & editing, Data curation, Investigation, Project administration, Visualization, Writing – original draft. PC: Data curation, Formal analysis, Methodology, Validation, Writing – review & editing, Resources. ÁP-G: Formal analysis, Methodology, Writing – review & editing. CP: Formal analysis, Writing – review & editing, Supervision. CS: Formal analysis, Supervision, Writing – review & editing, Conceptualization, Investigation, Methodology, Resources, Validation.

## References

[ref1] Agarbati A. Canonico L. Marini E. Zannini E. Ciani M. Comitini F. (2020). Potential probiotic yeasts sourced from natural environmental and spontaneous processed foods. Food Secur. 9:287. doi: 10.3390/foods9030287 PMC7143343 32143376

[ref2] Amorim J. C. Piccoli R. H. Duarte W. F. (2018). Probiotic potential of yeasts isolated from pineapple and their use in the elaboration of potentially functional fermented beverages. Food Res. Int. 107, 518–527. doi: 10.1016/j.foodres.2018.02.054, PMID: 29580515

[ref3] Andrade R. P. Oliveira D. R. Lopes A. C. A. de Abreu L. R. Duarte W. F. (2019). Survival of *Kluyveromyces lactis* and *Torulaspora delbrueckii* to simulated gastrointestinal conditions and their use as single and mixed inoculum for cheese production. Food Res. Int. 125:108620. doi: 10.1016/j.foodres.2019.108620, PMID: 31554038

[ref4] Basavaiah R. Nagesh M. Harishchandra Sripathy M. Vardhan Batra H. (2019). In vitro screening and characterization of kefir yeast for probiotic attributes. Int. J. Food Sci. 5, 1–11.

[ref5] Begley M. Hill C. Gahan C. G. M. (2006). Bile salt hydrolase activity in probiotics. Appl. Environ. Microbiol. 72, 1729–1738. doi: 10.1128/AEM.72.3.1729-1738.2006, PMID: 16517616 PMC1393245

[ref6] Binda S. Hill C. Johansen E. Obis D. Pot B. Sanders M. E.. (2020). Criteria to qualify microorganisms as “probiotic” in foods and dietary supplements. Front. Microbiol. 11:1662. doi: 10.3389/fmicb.2020.01662, PMID: 32793153 PMC7394020

[ref7] Cardoso R. R. Neto R. O. dos Santos D'Almeida C. T. do Nascimento T. P. Pressete C. G. Azevedo L.. (2020). Kombuchas from green and black teas have different phenolic profile, which impacts their antioxidant capacities, antibacterial and antiproliferative activities. Food Res. Int. 128:108782. doi: 10.1016/j.foodres.2019.108782, PMID: 31955755

[ref8] Cho Y. J. Kim D. H. Jeong D. Seo K. H. Jeong H. S. Lee H. G.. (2018). Characterization of yeasts isolated from kefir as a probiotic and its synergic interaction with the wine byproduct grape seed flour/extract. LWT 90, 535–539. doi: 10.1016/j.lwt.2018.01.010

[ref9] Chuah L. O. Foo H. L. Loh T. C. Mohammed Alitheen N. B. Yeap S. K. Abdul Mutalib N. E.. (2019). Postbiotic metabolites produced by *Lactobacillus plantarum* strains exert selective cytotoxicity effects on cancer cells. BMC Complement. Altern. Med. 19:114. doi: 10.1186/s12906-019-2528-2 31159791 PMC6547513

[ref10] Crauwels S. Zhu B. Steensels J. Busschaert P. de Samblanx G. Marchal K.. (2014). Assessing genetic diversity among *Brettanomyces* yeasts by DNA fingerprinting and whole-genome sequencing. Appl. Environ. Microbiol. 80, 4398–4413. doi: 10.1128/AEM.00601-14, PMID: 24814796 PMC4068659

[ref11] Czerucka D. Piche T. Rampal P. (2007). Review article: yeast as probiotics—*Saccharomyces boulardii*. Aliment. Pharmacol. Ther. 26, 767–778. doi: 10.1111/j.1365-2036.2007.03442.x 17767461

[ref12] de Melo Pereira G. V. de Oliveira Coelho B. Magalhães Júnior A. I. Thomaz-Soccol V. Soccol C. R. (2018). How to select a probiotic? A review and update of methods and criteria. Biotechnol. Adv. 36, 2060–2076. doi: 10.1016/j.biotechadv.2018.09.003 30266342

[ref13] de Miranda J. F. Ruiz L. F. Silva C. B. Uekane T. M. Silva K. A. Gonzalez A. G. M.. (2022). Kombucha: a review of substrates, regulations, composition, and biological properties. J. Food Sci. 87, 503–527. doi: 10.1111/1750-3841.16029, PMID: 35029317

[ref14] Del Re B. Sgorbati B. Miglioli M. Palenzona D. (2000). Adhesion, autoaggregation and hydrophobicity of 13 strains of *Bifidobacterium longum*. Lett. Appl. Microbiol. 31, 438–442. doi: 10.1046/j.1365-2672.2000.00845.x, PMID: 11123552

[ref15] Delesa D. A. (2017). Overview of anticancer activity of lactic acid bacteria international journal of advanced research in biological sciences overview of anticancer activity of lactic acid bacteria. Int. J. Adv. Res. Biol. Sci. 4, 166–177. doi: 10.22192/ijarbs.2017.04.12.017

[ref16] Di Cagno R. Filannino P. Cantatore V. Polo A. Celano G. Martinovic A.. (2020). Design of potential probiotic yeast starters tailored for making a cornelian cherry (*Cornus mas L.*) functional beverage. Int. J. Food Microbiol. 323:108591. doi: 10.1016/j.ijfoodmicro.2020.108591, PMID: 32222654

[ref17] Diez-Ozaeta I. Astiazaran O. J. (2022). Recent advances in Kombucha tea: microbial consortium, chemical parameters, health implications and biocellulose production. Int. J. Food Microbiol. 377:109783. doi: 10.1016/j.ijfoodmicro.2022.109783, PMID: 35728418

[ref18] EFSA-BIOHAZ Panel (2007). Opinion of the scientific committee on a request from EFSA on the introduction of a qualified presumption of safety (QPS) approach for assessment of selected microorganisms referred to EFSA. EFSA J. 5, 1–16. doi: 10.2903/j.efsa.2007.587

[ref19] Esteve-Zarzoso B. Belloch C. Uruburul F. Querol A. (1999). Identification of yeasts by RFLP analysis of the 5.85 rRNA gene and the two ribosomal internal transcribed spacers. Int. J. Syst. Evol. Microbiol. 49, 329–337. doi: 10.1099/00207713-49-1-329, PMID: 10028278

[ref20] Fadda M. E. Mossa V. Deplano M. Pisano M. B. Cosentino S. (2017). In vitro screening of *Kluyveromyces* strains isolated from Fiore Sardo cheese for potential use as probiotics. LWT 75, 100–106. doi: 10.1016/j.lwt.2016.08.020

[ref21] Fernández-Pacheco P. Ramos Monge I. M. Fernández-González M. Poveda Colado J. M. Arévalo-Villena M. (2021). Safety evaluation of yeasts with probiotic potential. Front. Nutr. 8:328. doi: 10.3389/fnut.2021.659328, PMID: 34095190 PMC8175779

[ref22] Food and Agriculture Organization of the United Nations and World Health Organization. (2006). Probiotics in food: health and nutritional properties and guidelines for evaluation. In: *FAO Food and Nutritional Paper No. 85*. Rome: Food and Agriculture Organization of the United Nations, World Health Organization, ISBN 92–5–105513-105510.

[ref23] Ghosh S. Bagheri B. Morgan H. H. Divol B. Setati M. E. (2015). Assessment of wine microbial diversity using ARISA and cultivation-based methods. Ann. Microbiol. 65, 1833–1840. doi: 10.1007/s13213-014-1021-x

[ref24] Gil-Rodríguez A. M. Carrascosa A. Requena T. (2015). Yeasts in foods and beverages: In vitro characterization of probiotic traits. LWT 64, 1156–1162. doi: 10.1016/j.lwt.2015.07.042

[ref25] Goktas H. Dikmen H. Demirbas F. Sagdic O. Dertli E. (2021). Characterisation of probiotic properties of yeast strains isolated from kefir samples. Int. J. Dairy Technol. 74, 715–722. doi: 10.1111/1471-0307.12802

[ref26] Goldenberg J. Z. Yap C. Lytvyn L. Lo C. K. F. Beardsley J. Mertz D.. (2017). Probiotics for the prevention of *Clostridium difficile*-associated diarrhea in adults and children. Cochrane Database Syst. Rev. 2017:CD006095. doi: 10.1002/14651858.CD006095.pub4, PMID: 29257353 PMC6486212

[ref27] Gomes B. C. Esteves C. T. Palazzo I. C. V. Darini A. L. C. Felis G. E. Sechi L. A.. (2008). Prevalence and characterization of *Enterococcus* spp. isolated from Brazilian foods. Food Microbiol. 25, 668–675. doi: 10.1016/j.fm.2008.03.008, PMID: 18541165

[ref28] Gut A. M. Vasiljevic T. Yeager T. Donkor O. N. (2019). Characterization of yeasts isolated from traditional kefir grains for potential probiotic properties. J. Funct. Foods 58, 56–66. doi: 10.1016/j.jff.2019.04.046

[ref29] Han S. Lu Y. Xie J. Fei Y. Zheng G. Wang Z.. (2021). Probiotic gastrointestinal transit and colonization after oral administration: a long journey. Front. Cell. Infect. Microbiol. 11:722. doi: 10.3389/fcimb.2021.609722, PMID: 33791234 PMC8006270

[ref30] Helmy E. A. Soliman S. A. Abdel-Ghany T. M. Ganash M. (2019). Evaluation of potentially probiotic attributes of certain dairy yeast isolated from buffalo sweetened Karish cheese. Heliyon. 5:e01649. doi: 10.1016/j.heliyon.2019.e01649, PMID: 31193166 PMC6520606

[ref31] Hye-Youn Y. Dong-Hyeon K. Hyeon-Jin K. Dongryeoul B. Kwang-Young S. Hyunsook K.. (2022). Survivability of *Kluyveromyces marxianus* isolated from Korean kefir in a simulated gastrointestinal environment. Front. Microbiol. 13:2097. doi: 10.3389/fmicb.2022.842097, PMID: 35283845 PMC8908258

[ref32] Ilango S. Antony U. (2021). Probiotic microorganisms from non-dairy traditional fermented foods. Trends Food Sci. Technol. 118, 617–638. doi: 10.1016/j.tifs.2021.05.034

[ref33] Jaehrig S. C. Rohn S. Kroh L. W. Fleischer L. G. Kurz T. (2007). In vitro potential antioxidant activity of (1-->3),(1-->6)-beta-D-glucan and protein fractions from *Saccharomyces cerevisiae* cell walls. J. Agric. Food Chem. 55, 4710–4716. doi: 10.1021/jf063209q 17516653

[ref34] Kapp J. M. Sumner W. (2019). Kombucha: a systematic review of the empirical evidence of human health benefit. Ann. Microbiol. 30, 66–70. doi: 10.1016/j.annepidem.2018.11.001, PMID: 30527803

[ref35] Kapteyn J. C. Ter Riet B. Vink E. Blad S. De Nobel H. Van Den Ende H.. (2001). Low external pH induces HOG1-dependent changes in the organization of the *Saccharomyces cerevisiae* cell wall. Mol. Microbiol. 39, 469–480. doi: 10.1046/j.1365-2958.2001.02242.x, PMID: 11136466

[ref36] Kumar M. Kumar A. Nagpal R. Mohania D. Behare P. Verma V.. (2010). Cancer-preventing attributes of probiotics: an update. Int. J. Food Nutr. Sci. 61, 473–496. doi: 10.3109/09637480903455971, PMID: 20187714

[ref37] Kunyeit L. Kurrey N. K. Anu-Appaiah K. A. Rao R. P. (2019). Probiotic yeasts inhibit virulence of non-*albicans Candida* species. MBio 10:10(5). doi: 10.1128/mBio.02307-19, PMID: 31615960 PMC6794482

[ref38] Kurtzman C. P. Robnett C. J. (1998). Identification and phylogeny of *ascomycetous* yeasts from analysis of nuclear large subunit (26S) ribosomal DNA partial sequences. Antonie Van Leeuwenhoek 73:8817. doi: 10.1023/A:1001761008817 9850420

[ref39] Lata P. Kumari R. Sharma K. B. Rangra S. Savitri S. (2022). In vitro evaluation of probiotic potential and enzymatic profiling of *Pichia kudriavzevii* Y33 isolated from traditional home-made mango pickle. J. Genet. Eng. Biotechnol. 20:132. doi: 10.1186/s43141-022-00416-2, PMID: 36083419 PMC9463414

[ref40] Latif A. Shehzad A. Niazi S. Zahid A. Ashraf W. Iqbal M. W.. (2023). Probiotics: mechanism of action, health benefits and their application in food industries. Front. Microbiol. 14:6674. doi: 10.3389/fmicb.2023.1216674, PMID: 37664108 PMC10470842

[ref41] Lavelle A. Hill C. (2019). Gut microbiome in health and disease: emerging diagnostic opportunities. Gastroenterol. Clin. North Am. 48, 221–235. doi: 10.1016/j.gtc.2019.02.003 31046972

[ref42] Liong M. T. Shah N. P. (2005). Bile salt deconjugation ability, bile salt hydrolase activity and cholesterol co-precipitation ability of lactobacilli strains. Int. Dairy J. 15, 391–398. doi: 10.1016/j.idairyj.2004.08.007

[ref43] Luang-In V. Saengha W. Karirat T. Buranrat B. Nudmamud-Thanoi S. Ma N. L.. (2020). Cytotoxic effects of *Saccharomyces cerevisiae*tc6 and *Lactobacillus brevis* TBRC 3003 isolated from Thai fermented foods. Trop. J. Pharm. Res. 19, 2385–2393. doi: 10.4314/tjpr.v19i11.20

[ref44] Lucena R. M. Dolz-Edo L. Brul S. de Morais M. A. Smits G. (2020). Extreme low cytosolic pH is a signal for cell survival in acid stressed yeast. Genes. 11, 1–25. doi: 10.3390/genes11060656, PMID: 32560106 PMC7349538

[ref45] Meneely P. M. Dahlberg C. L. Rose J. K. (2019). Working with worms: *Caenorhabditis elegans* as a model organism. Curr Protocols Essent Lab Techn 19:e35. doi: 10.1002/cpet.35

[ref46] Menezes A. G. T. Ramos C. L. Cenzi G. Melo D. S. Dias D. R. Schwan R. F. (2020). Probiotic potential, antioxidant activity, and phytase production of indigenous yeasts isolated from indigenous fermented foods. Probiot. Antimicrob. Protein. 12, 280–288. doi: 10.1007/s12602-019-9518-z, PMID: 30685824

[ref47] Miguel G. A. Carlsen S. Arneborg N. Saerens S. M. G. Laulund S. Knudsen G. M. (2022). Non-Saccharomyces yeasts for beer production: insights into safety aspects and considerations. Int. J. Food Microbiol. 383:109951. doi: 10.1016/j.ijfoodmicro.2022.109951, PMID: 36240605

[ref48] Miot-Sertier C. Lonvaud-Funel A. (2007). Development of a molecular method for the typing of *Brettanomyces bruxellensis* (*Dekkera bruxellensis*) at the strain level. J. Appl. Microbiol. 102, 555–562. doi: 10.1111/j.1365-2672.2006.03069.x, PMID: 17241362

[ref49] Moré M. I. Swidsinski A. (2015). *Saccharomyces boulardii* CNCM I-745 supports regeneration of the intestinal microbiota after diarrheic dysbiosis—a review. Clin. Exp. Gastroenterol. 8, 237–255. doi: 10.2147/CEG.S85574, PMID: 26316791 PMC4542552

[ref50] Murzyn A. Krasowska A. Stefanowicz P. Dziadkowiec D. Łukaszewicz M. (2010). Capric acid secreted by *S. Boulardii* inhibits *C. albicans* filamentous growth, adhesion and biofilm formation. PLoS One 5:e12050. doi: 10.1371/journal.pone.0012050, PMID: 20706577 PMC2919387

[ref51] Naimah A. K. Al-Manhel A. J. A. Al-Shawi M. J. (2018). Isolation, purification and characterization of antimicrobial peptides produced from *Saccharomyces boulardii*. Int. J. Pept. Res. Ther. 24, 455–461. doi: 10.1007/s10989-017-9632-2

[ref52] Neut C. Mahieux S. Dubreuil L. J. (2017). Antibiotic susceptibility of probiotic strains: is it reasonable to combine probiotics with antibiotics? Med. Mal. Infect. 47, 477–483. doi: 10.1016/j.medmal.2017.07.001, PMID: 28797834

[ref53] Noriega L. Cuevas I. Margolles A. de los Reyes-Gavilán C. G. (2006). Deconjugation and bile salts hydrolase activity by *Bifidobacterium* strains with acquired resistance to bile. Int. Dairy J. 16, 850–855. doi: 10.1016/j.idairyj.2005.09.008

[ref54] Ntiantiasi N. Lianou A. (2023). Isolation and *in vitro* screening of the probiotic potential of microorganisms from fermented food products. Front. Ind. Microbiol 1:483. doi: 10.3389/finmi.2023.1257483

[ref55] O’Toole P. W. Marchesi J. R. Hill C. (2017). Next-generation probiotics: the spectrum from probiotics to live biotherapeutics. Nat. Microbiol. 2:17057. doi: 10.1038/nmicrobiol.2017.57, PMID: 28440276

[ref56] Ochangco H. S. Gamero A. Smith I. M. Christensen J. E. Jespersen L. Arneborg N. (2016). In vitro investigation of *Debaryomyces hansenii* strains for potential probiotic properties. World J. Microbiol. Biotechnol. 32:141. doi: 10.1007/s11274-016-2109-1, PMID: 27430508

[ref57] Ogunremi O. R. Sanni A. I. Agrawal R. (2015). Probiotic potentials of yeasts isolated from some cereal-based Nigerian traditional fermented food products. J. Appl. Microbiol. 119, 797–808. doi: 10.1111/jam.12875, PMID: 26095794

[ref58] Owusu-Kwarteng J. Tano-Debrah K. Akabanda F. Jespersen L. (2015). Technological properties and probiotic potential of *Lactobacillus fermentum* strains isolated from west African fermented millet dough applied microbiology. BMC Microbiol. 15:261. doi: 10.1186/s12866-015-0602-6, PMID: 26560346 PMC4642623

[ref59] Pakbin B. Dibazar S. P. Allahyari S. Javadi M. Amani Z. Farasat A.. (2022). Anticancer properties of probiotic *Saccharomyces boulardii* supernatant on human breast cancer cells. Probiotics Antimicrob. Proteins. 14, 1130–1138. doi: 10.1007/s12602-021-09756-w, PMID: 35094296

[ref60] Panicker A. S. Ali S. A. Anand S. Panjagari N. R. Kumar S. Mohanty A. K.. (2018). Evaluation of some in vitro probiotic properties of *Lactobacillus fermentum* strains. J. Food Sci. Technol. 55, 2801–2807. doi: 10.1007/s13197-018-3197-8, PMID: 30042597 PMC6033835

[ref61] Pennacchia C. Blaiotta G. Pepe O. Villani F. (2008). Isolation of *Saccharomyces cerevisiae* strains from different food matrices and their preliminary selection for a potential use as probiotics. J. Appl. Microbiol. 105, 1919–1928. doi: 10.1111/j.1365-2672.2008.03968.x, PMID: 19120638

[ref62] Pham T. Wimalasena T. Box W. G. Koivuranta K. Storgårds E. Smart K. A.. (2011). Evaluation of ITS PCR and RFLP for differentiation and identification of brewing yeast and brewery “wild” yeast contaminants. J. Inst. Brewing 117, 556–568. doi: 10.1002/j.2050-0416.2011.tb00504.x, PMID: 32834175 PMC7197508

[ref63] Pihurov M. Păcularu-Burada B. Cotârleţ M. Vasile M. A. Bahrim G. E. (2021). Novel insights for metabiotics production by using artisanal probiotic cultures. Microorganisms. 9:184. doi: 10.3390/microorganisms9112184, PMID: 34835310 PMC8624174

[ref64] Pisano M. B. Viale S. Conti S. Fadda M. E. Deplano M. Melis M. P.. (2014). Preliminary evaluation of probiotic properties of *Lactobacillus* strains isolated from Sardinian dairy products. Biomed. Res. Int. 2014:390. doi: 10.1155/2014/286390, PMID: 25054135 PMC4099116

[ref65] Poupet C. Saraoui T. Veisseire P. Bonnet M. Dausset C. Gachinat M.. (2019). *Lactobacillus rhamnosus* Lcr35 as an effective treatment for preventing *Candida albicans* infection in the invertebrate model *Caenorhabditis elegans*: first mechanistic insights. PLoS One 14:e0216184. doi: 10.1371/journal.pone.0216184, PMID: 31693670 PMC6834333

[ref66] Ragavan M. L. Das N. (2020). *In vitro* studies on therapeutic potential of probiotic yeasts isolated from various sources. Curr. Microbiol. 77, 2821–2830. doi: 10.1007/s00284-020-02100-5, PMID: 32591923

[ref67] Roselli M. Schifano E. Guantario B. Zinno P. Uccelletti D. Devirgiliis C. (2019). *Caenorhabditis elegans* and probiotics interactions from a prolongevity perspective. Int. J. Mol. Sci. 20:5020. doi: 10.3390/ijms20205020, PMID: 31658751 PMC6834311

[ref68] Saadat Y. R. Khosroushahi A. Y. Movassaghpour A. A. Talebi M. Gargari B. P. (2020). Modulatory role of exopolysaccharides of *Kluyveromyces marxianus* and *Pichia kudriavzevii* as probiotic yeasts from dairy products in human colon cancer cells. J. Funct. Foods 64:103675. doi: 10.1016/j.jff.2019.103675

[ref69] Sakandar H. A. Usman K. Imran M. (2018). Isolation and characterization of gluten-degrading *Enterococcus mundtii* and *Wickerhamomyces anomalus*, potential probiotic strains from indigenously fermented sourdough (Khamir). LWT 91, 271–277. doi: 10.1016/j.lwt.2018.01.023

[ref70] Şanlidere Aloğlu H. Demir Özer E. Öner Z. (2016). Assimilation of cholesterol and probiotic characterization of yeast strains isolated from raw milk and fermented foods. Int. J. Dairy Technol. 69, 63–70. doi: 10.1111/1471-0307.12217

[ref71] Sharma M. Chandel D. Shukla G. (2020). Antigenotoxicity and cytotoxic potentials of metabiotics extracted from isolated probiotic, *Lactobacillus rhamnosus* MD 14 on Caco-2 and HT-29 human colon cancer cells. Nutr. Cancer 72, 110–119. doi: 10.1080/01635581.2019.1615514, PMID: 31266374

[ref72] Simões L. A. Cristina de Souza A. Ferreira I. Melo D. S. Lopes L. A. A. Magnani M.. (2021). Probiotic properties of yeasts isolated from Brazilian fermented table olives. J. Appl. Microbiol. 131, 1983–1997. doi: 10.1111/jam.15065, PMID: 33704882

[ref73] Sourabh A. Kanwar S. S. Sharma O. P. (2011). Screening of indigenous yeast isolates obtained from traditional fermented foods of Western Himalayas for probiotic attributes. J. Yeast Fungal Res. 2, 117–126. doi: 10.5897/JYFR.9000045

[ref74] Syal P. Vohra A. (2013). Probiotic potential of yeasts isolated from traditional fermented foods. Int. Microbiol. Res. 5, 390–398. doi: 10.9735/0975-5276.5.2.390-398

[ref75] Syal P. Vohra A. (2014). Probiotic attributes of yeast-like fungus Geotrichum klebahnii. Afr. J. Microbiol. Res. 8, 2037–2043. doi: 10.5897/AJMR2013.6175

[ref76] Veisseire P. Bonnet M. Saraoui T. Poupet C. Camarès O. Gachinat M.. (2020). Investigation into in vitro and in vivo *Caenorhabditis elegans* models to select cheese yeasts as probiotic candidates for their preventive effects against *Salmonella Typhimurium*. Microorganisms. 8, 1–16. doi: 10.3390/microorganisms8060922, PMID: 32570901 PMC7356738

[ref77] Villarreal-Soto S. A. Bouajila J. Pace M. Leech J. Cotter P. D. Souchard J. P.. (2020). Metabolome-microbiome signatures in the fermented beverage, Kombucha. Int. J. Food Microbiol. 333:108778. doi: 10.1016/j.ijfoodmicro.2020.108778, PMID: 32731153

[ref78] Zaouche A. Loukil C. de Lagausie P. Peuchmaur M. Macry J. Fitoussi F.. (2000). Effects of oral *Saccharomyces boulardii* on bacterial overgrowth, translocation, and intestinal adaptation after small-bowel resection in rats. Scand. J. Gastroenterol. 35, 160–165. doi: 10.1080/003655200750024326, PMID: 10720113

