# Supplementary material for: Potential probiotic and functional properties of Brettanomyces strains isolated from kombucha tea
Source: Front Microbiol. 2024 Jul 23;15:1415616. doi: 10.3389/fmicb.2024.1415616 (PMC11300377; doi:10.3389/fmicb.2024.1415616)
Supplement: Supplementary file 1 [file Data_Sheet_1.PDF]

Supplementary Material

1 Supplementary Figures and Tables

1.1 Supplementary Tables

Table 1. Molecular identification of the yeasts isolates by the sequence of the D1D2 region and the ITS RFLP analysis.

Isolate	Closest species (GeneBank Accesion number)	Identity (%)	ITS RFLP analysis (bp)[a]			ITS size (bp)[a]
			Hae III	Hinf I	Hha II (Cfo I)	
**UVI55**	*Brettanomyces bruxellensis* – strain CBS:74 (KY107614.1)	100	348, 102	249, 196	210, 121, 79	455
**UVI56**	*Brettanomyces bruxellensis* – strain CBS:74 (KY107614.1)	100	348, 102	249, 196	210, 121, 79	460
**UVI57**	*Brettanomyces anomalus* – strain CBS:4461 (KY107595.1)	99,84	383, 110	196, 196, 82	243, 127, 73	504
**UVI58**	*Brettanomyces anomalus* – strain CBS:4461 (KY107595.1)	99,67	383, 110	215, 215, 82	243, 127, 73	509

[a]base pair

1.2 Supplementary Figures

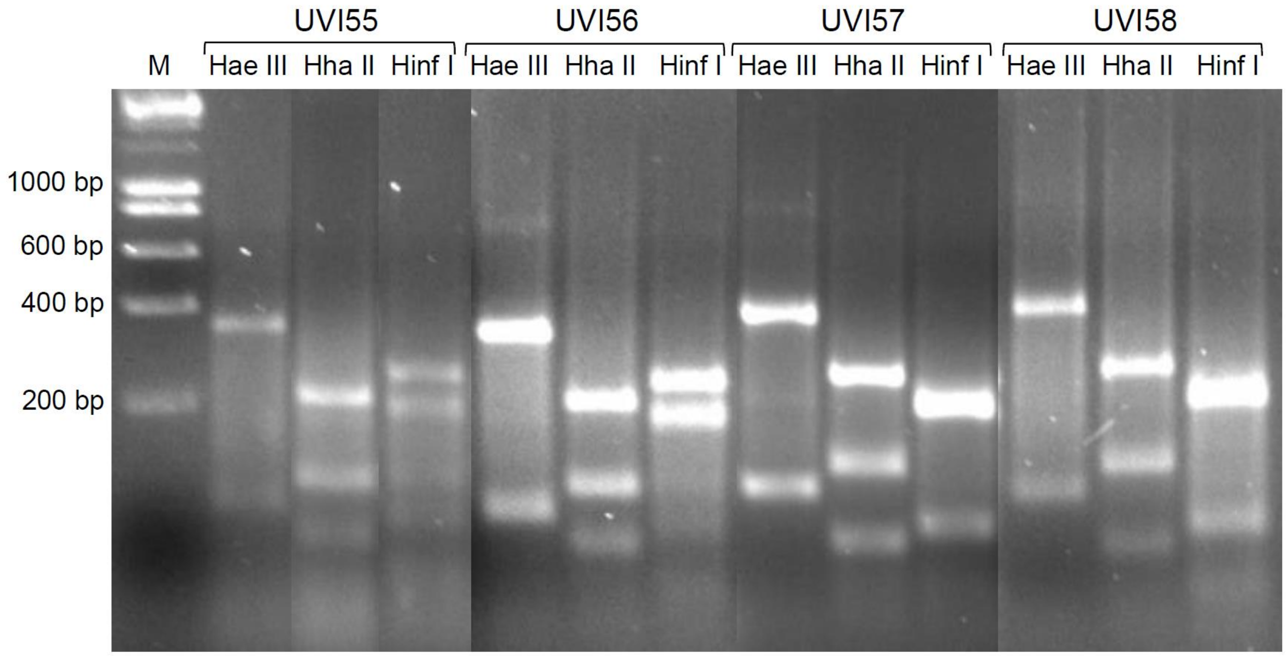

Figure 1. Restriction analysis of the ITS/5.85 ribosomal DNA region with the endonucleases *Hae*III, *Hha*II and *Hinf*I. Lane M corresponds to molecular size standard (NZYDNA Ladder III, nzytech).

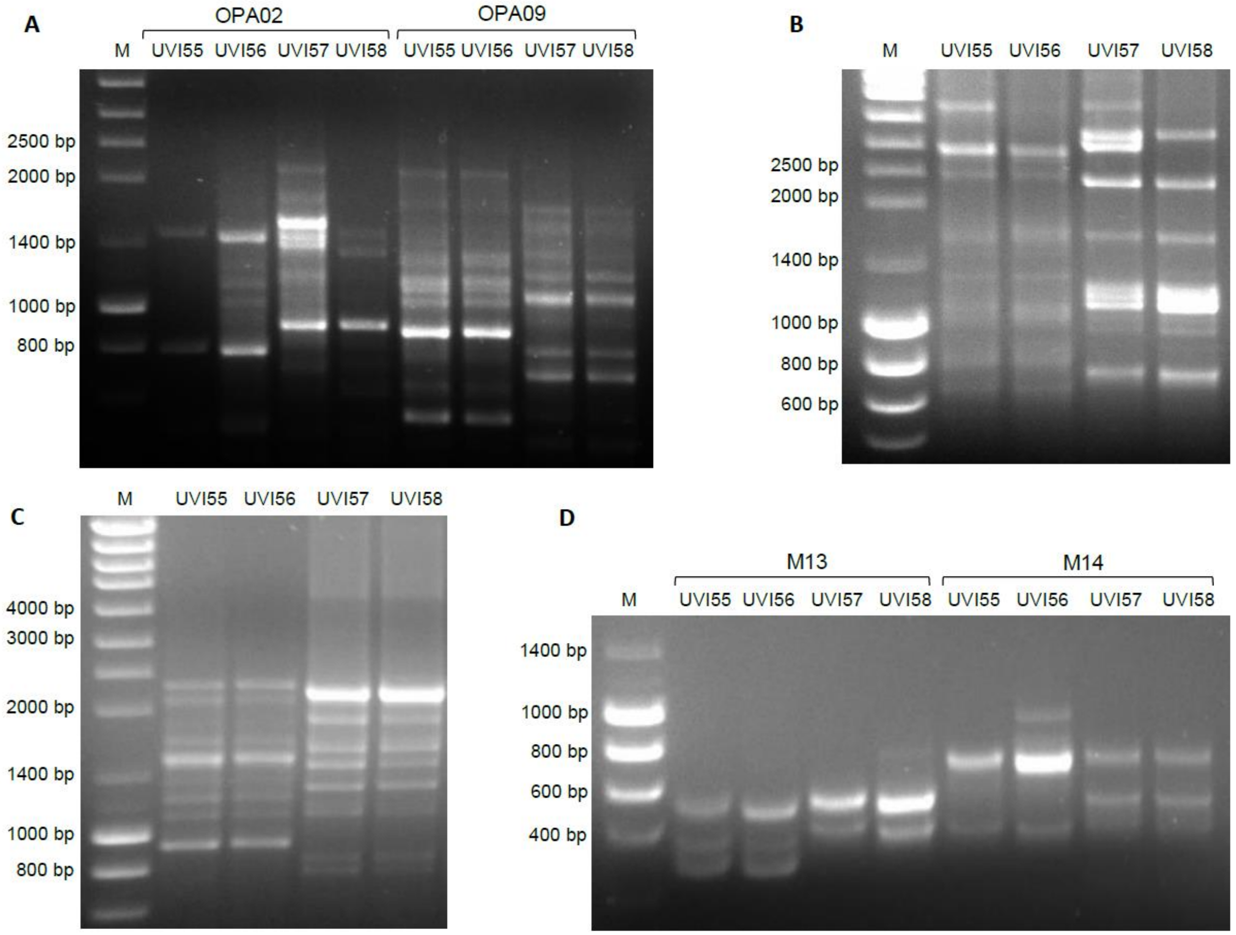

Figure 2. Random-amplified polymorphic DNA patterns of the yeast isolates generated with OPA02 and OPA09 (A), GTG_5 (B), GAC_5 (C), M13 and M14 (D). Lanes M correspond to molecular size standards (NZYDNA Ladder III, nzytech).
