# Supplementary material for: Potential probiotic and functional properties of Brettanomyces strains isolated from kombucha tea
Source: Front Microbiol. 2024 Jul 23;15:1415616. doi: 10.3389/fmicb.2024.1415616 (PMC11300377; doi:10.3389/fmicb.2024.1415616)
Supplement: Supplementary file 2 [file Data_Sheet_2.PDF]

*Supplementary Material*

## 1 Supplementary Data

## 2 Supplementary Figures and Tables

For more information on Supplementary Material and for details on the different file types accepted, please see here.

**Table 1**. Molecular identification of the yeasts isolates by the sequence of the D1D2 region and the ITS RFLP analysis.

| Isolate | Closest species (GeneBank Accesion number) | Identity (%) | ITS RFLP analysis (bp)[a] | | | ITS size (bp)[a] |
|---|---|---|---|---|---|---|
| | | | Hae III | Hinf I | Hha II (Cfo I) | |
| **UVI55** | *Brettanomyces bruxellensis* – strain CBS:74 (KY107614.1) | 100 | 348, 102 | 249, 196 | 210, 121, 79 | 455 |
| **UVI56** | *Brettanomyces bruxellensis* – strain CBS:74 (KY107614.1) | 100 | 348, 102 | 249, 196 | 210, 121, 79 | 460 |
| **UVI57** | *Brettanomyces anomalus* – strain CBS:4461 (KY107595.1) | 99,84 | 383, 110 | 196, 196, 82 | 243, 127, 73 | 504 |
| **UVI58** | *Brettanomyces anomalus* – strain CBS:4461 (KY107595.1) | 99,67 | 383, 110 | 215, 215, 82 | 243, 127, 73 | 509 |

[a]base pair

### 2.1 Supplementary Figures

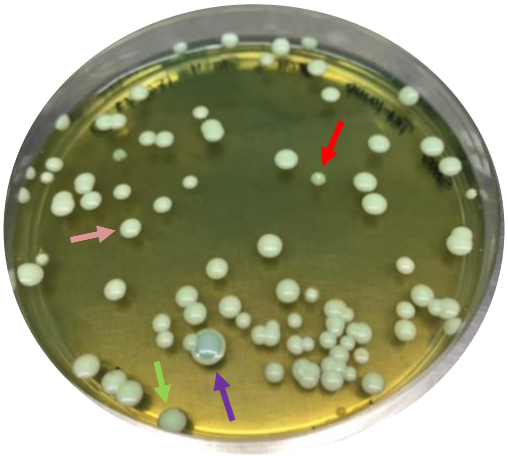

**Figure S1**. Kombucha tea sample plated on WL medium. Arrows indicate differentiated morphotypes.

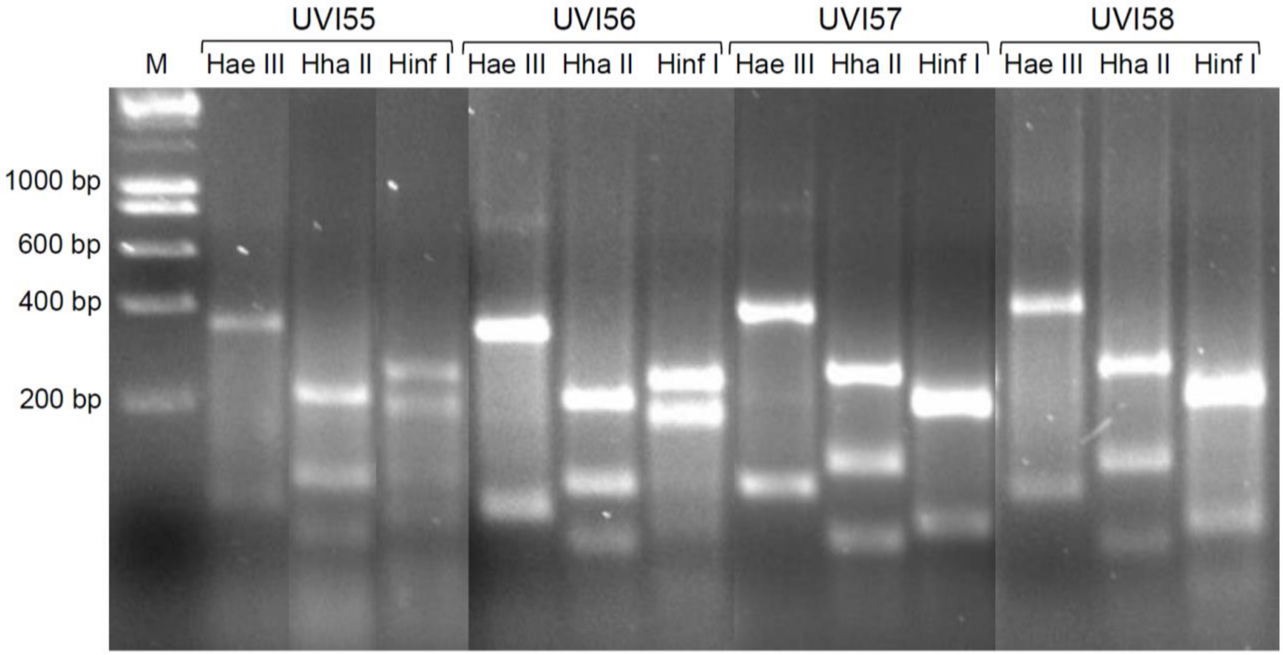


**Figure S2**. Restriction analysis of the ITS/5.85 ribosomal DNA region with the endonucleases *Hae*III, *Hha*II and *Hinf*I. Lane M corresponds to molecular size standard (NZYDNA Ladder III, nzytech).

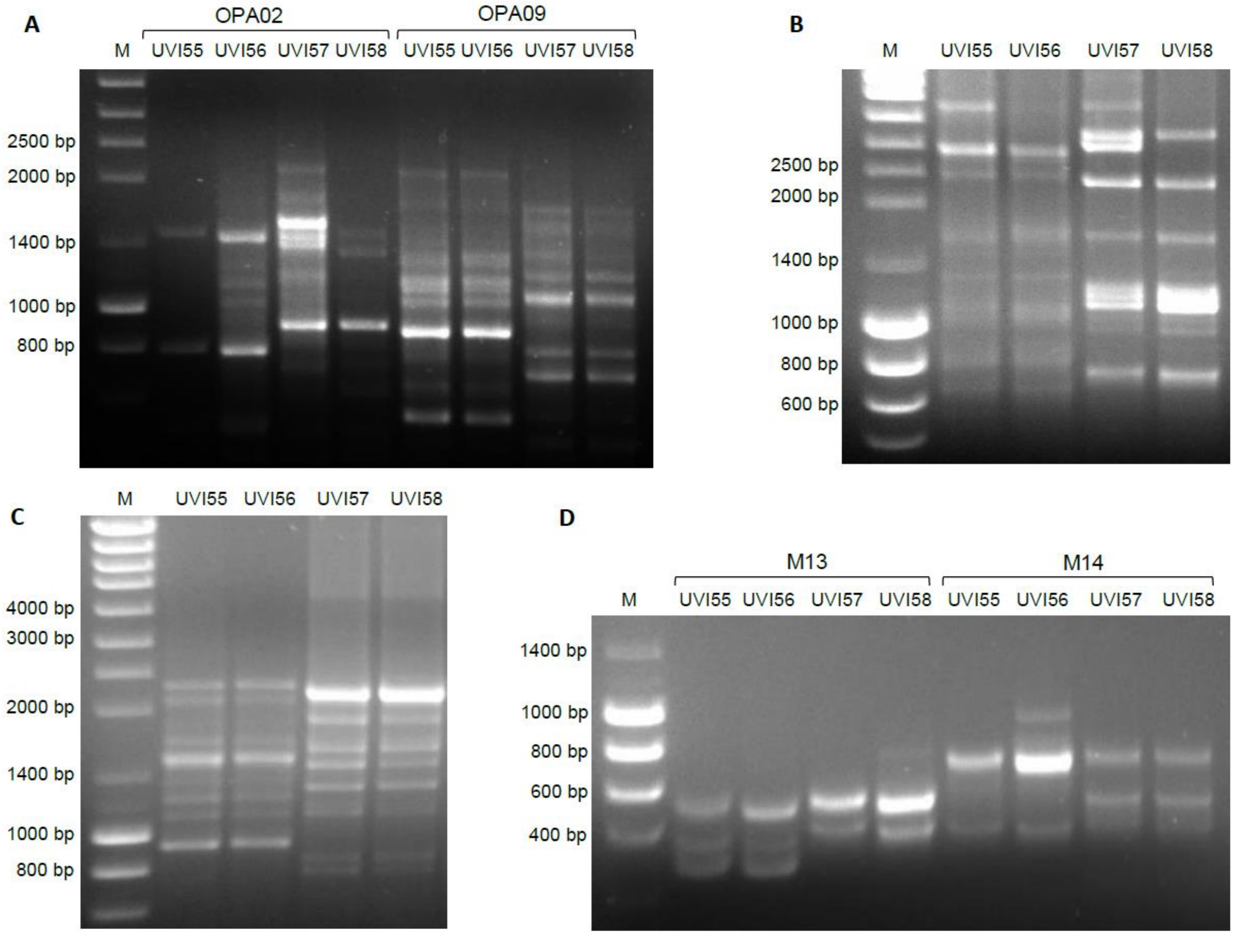


**Figure S3**. Random-amplified polymorphic DNA patterns of the yeast isolates generated with OPA02 and OPA09 (A), $GTG_5$ (B), $GAC_5$ (C), M13 and M14 (D). Lanes M correspond to molecular size standards (NZYDNA Ladder III, nzytech).

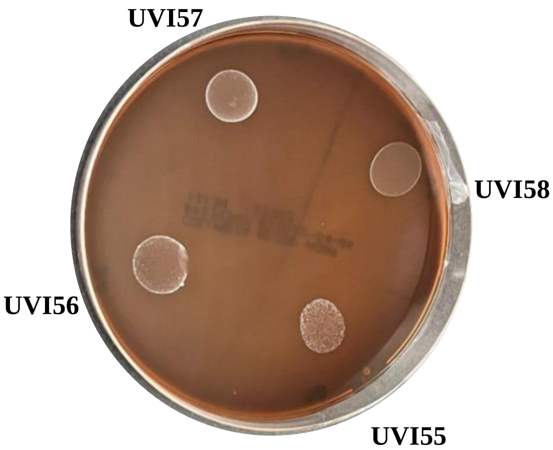


**Figure S4**. Hemolytic activity of yeasts UVI55, UVI56, UVI57 and UVI58.

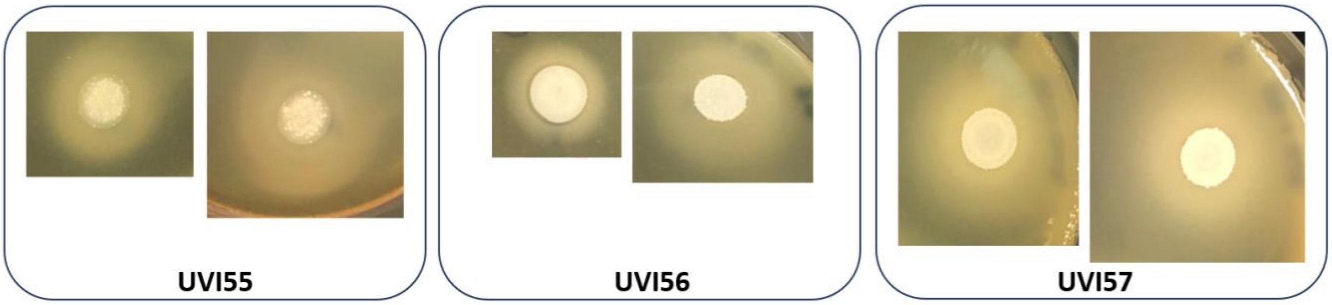


**Figure S5**. Bile Salt hydrolase activity of yeasts UVI55, UVI56 and UVI57 after 48 hours at 30°C (left colonies) and after 6 days at 30°C (right colonies).

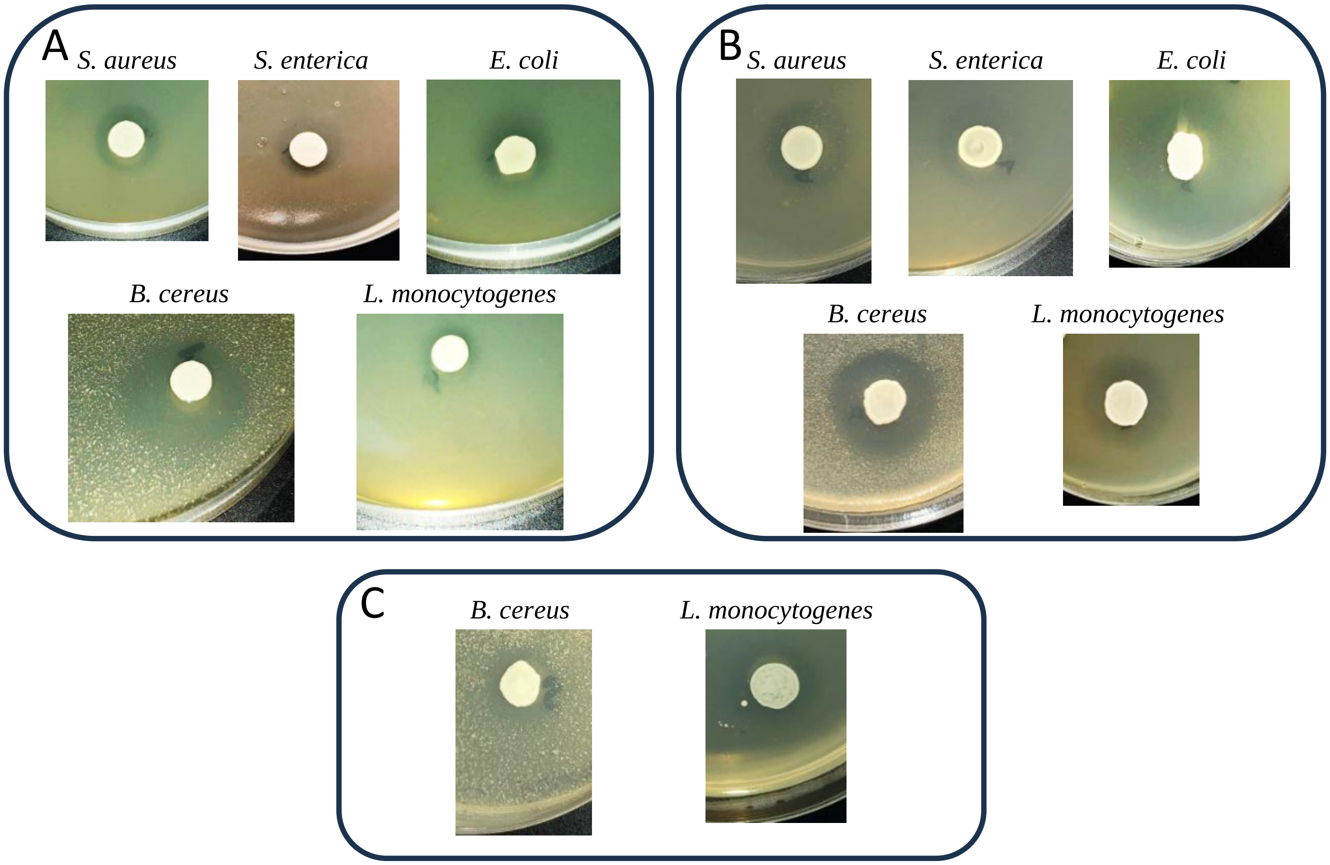


**Figure S6**. Antimicrobial activity of yeasts strains UVI55 (A), UVI56 (B) and UVI57 (C), against different pathogens.
