# Supplementary material for: Potential probiotic and functional properties of Brettanomyces strains isolated from kombucha tea
Source: Front Microbiol. 2024 Jul 23;15:1415616. doi: 10.3389/fmicb.2024.1415616 (PMC11300377; doi:10.3389/fmicb.2024.1415616)
Supplement: Supplementary file 3 [file Table_1.DOCX]

Supplementary Material

# Supplementary Figures and Tables

## Supplementary Tables

**Table 1**. Molecular identification of the yeasts isolates by the sequence of the D1D2 region and the ITS RFLP analysis.

| **Isolate** | **Closest species (GeneBank Accesion number)** | **Identity (%)** | **ITS RFLP analysis (bp)^a^** | | | **ITS size (bp)^a^** |
| --- | --- | --- | --- | --- | --- | --- |
|  | | | **Hae III** | **Hinf I** | **Hha II (Cfo I)** |  |
| **UVI55** | *Brettanomyces bruxellensis* – strain CBS:74 (KY107614.1) | 100 | 348, 102 | 249, 196 | 210, 121, 79 | 455 |
| **UVI56** | *Brettanomyces bruxellensis* – strain CBS:74 (KY107614.1) | 100 | 348, 102 | 249, 196 | 210, 121, 79 | 460 |
| **UVI57** | *Brettanomyces anomalus* – strain CBS:4461 (KY107595.1) | 99,84 | 383, 110 | 196, 196, 82 | 243, 127, 73 | 504 |
| **UVI58** | *Brettanomyces anomalus* – strain CBS:4461 (KY107595.1) | 99,67 | 383, 110 | 215, 215, 82 | 243, 127, 73 | 509 |

^a^base pair

## Supplementary Figures


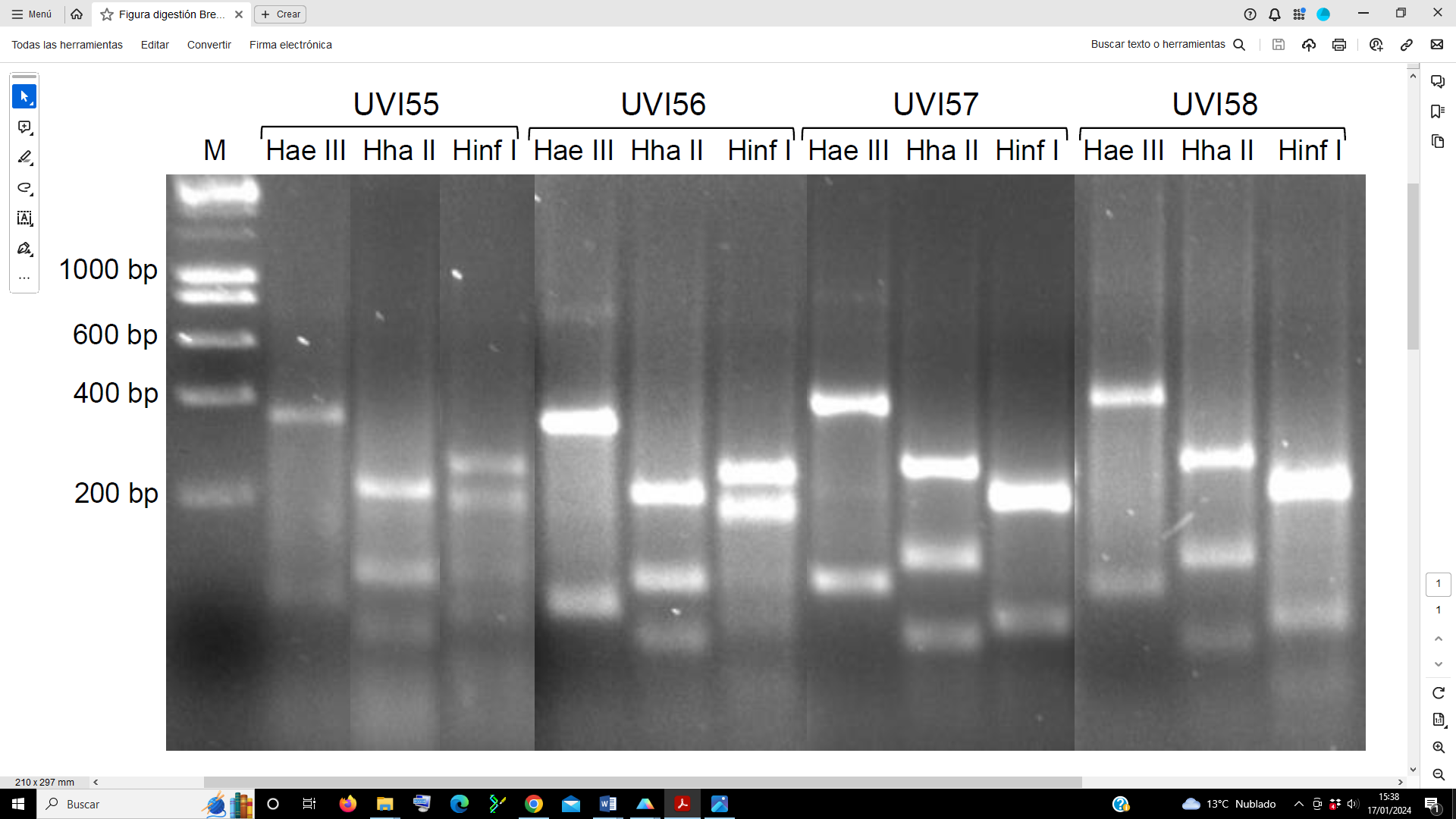


**Figure 1**. Restriction analysis of the ITS/5.85 ribosomal DNA region with the endonucleases *Hae*III, *Hha*II and *Hinf*I. Lane M corresponds to molecular size standard (NZYDNA Ladder III, nzytech).


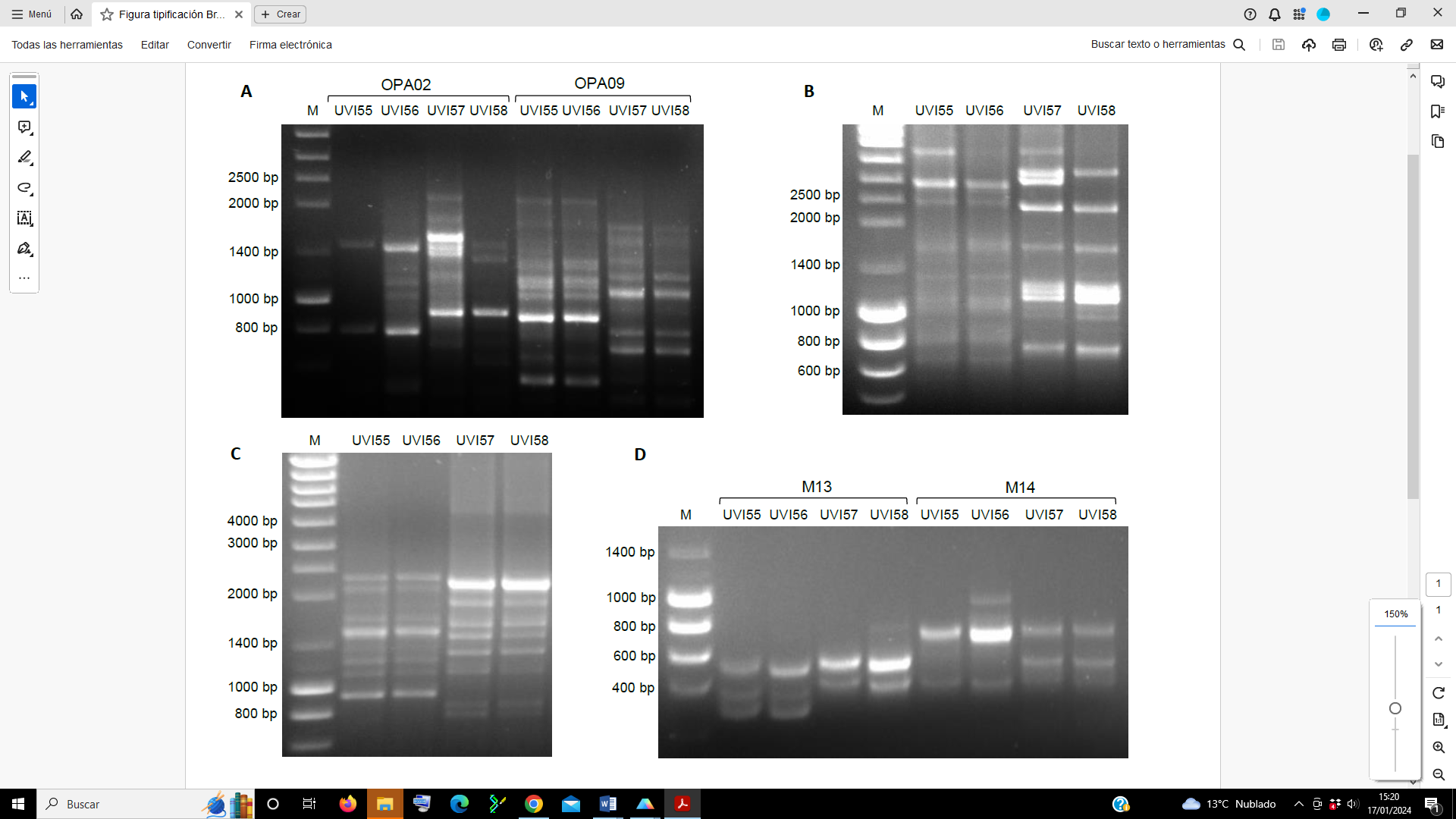


**Figure 2**. Random-amplified polymorphic DNA patterns of the yeast isolates generated with OPA02 and OPA09 (A), GTG_5_ (B), GAC_5_ (C), M13 and M14 (D). Lanes M correspond to molecular size standards (NZYDNA Ladder III, nzytech).
