# Supplementary material for: Potential probiotic and functional properties of Brettanomyces strains isolated from kombucha tea
Source: Front Microbiol. 2024 Jul 23;15:1415616. doi: 10.3389/fmicb.2024.1415616 (PMC11300377; doi:10.3389/fmicb.2024.1415616)

Supplementary Material

# Supplementary Data

# Supplementary Figures and Tables

For more information on Supplementary Material and for details on the different file types accepted, please see [here](https://www.frontiersin.org/guidelines/author-guidelines#supplementary-material).

**Table 1**. Molecular identification of the yeasts isolates by the sequence of the D1D2 region and the ITS RFLP analysis.

| **Isolate** | **Closest species (GeneBank Accesion number)** | **Identity (%)** | **ITS RFLP analysis (bp)^a^** | | | **ITS size (bp)^a^** |
| --- | --- | --- | --- | --- | --- | --- |
|  | | | **Hae III** | **Hinf I** | **Hha II (Cfo I)** |  |
| **UVI55** | *Brettanomyces bruxellensis* – strain CBS:74 (KY107614.1) | 100 | 348, 102 | 249, 196 | 210, 121, 79 | 455 |
| **UVI56** | *Brettanomyces bruxellensis* – strain CBS:74 (KY107614.1) | 100 | 348, 102 | 249, 196 | 210, 121, 79 | 460 |
| **UVI57** | *Brettanomyces anomalus* – strain CBS:4461 (KY107595.1) | 99,84 | 383, 110 | 196, 196, 82 | 243, 127, 73 | 504 |
| **UVI58** | *Brettanomyces anomalus* – strain CBS:4461 (KY107595.1) | 99,67 | 383, 110 | 215, 215, 82 | 243, 127, 73 | 509 |

^a^base pair

## Supplementary Figures


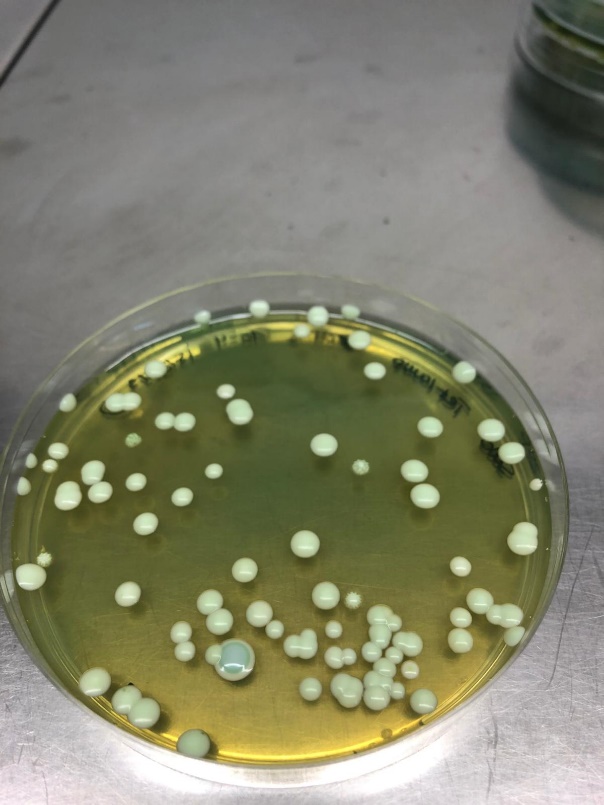


**Figure S1**. Kombucha tea sample plated on WL medium. Arrows indicate differentiated morphotypes.


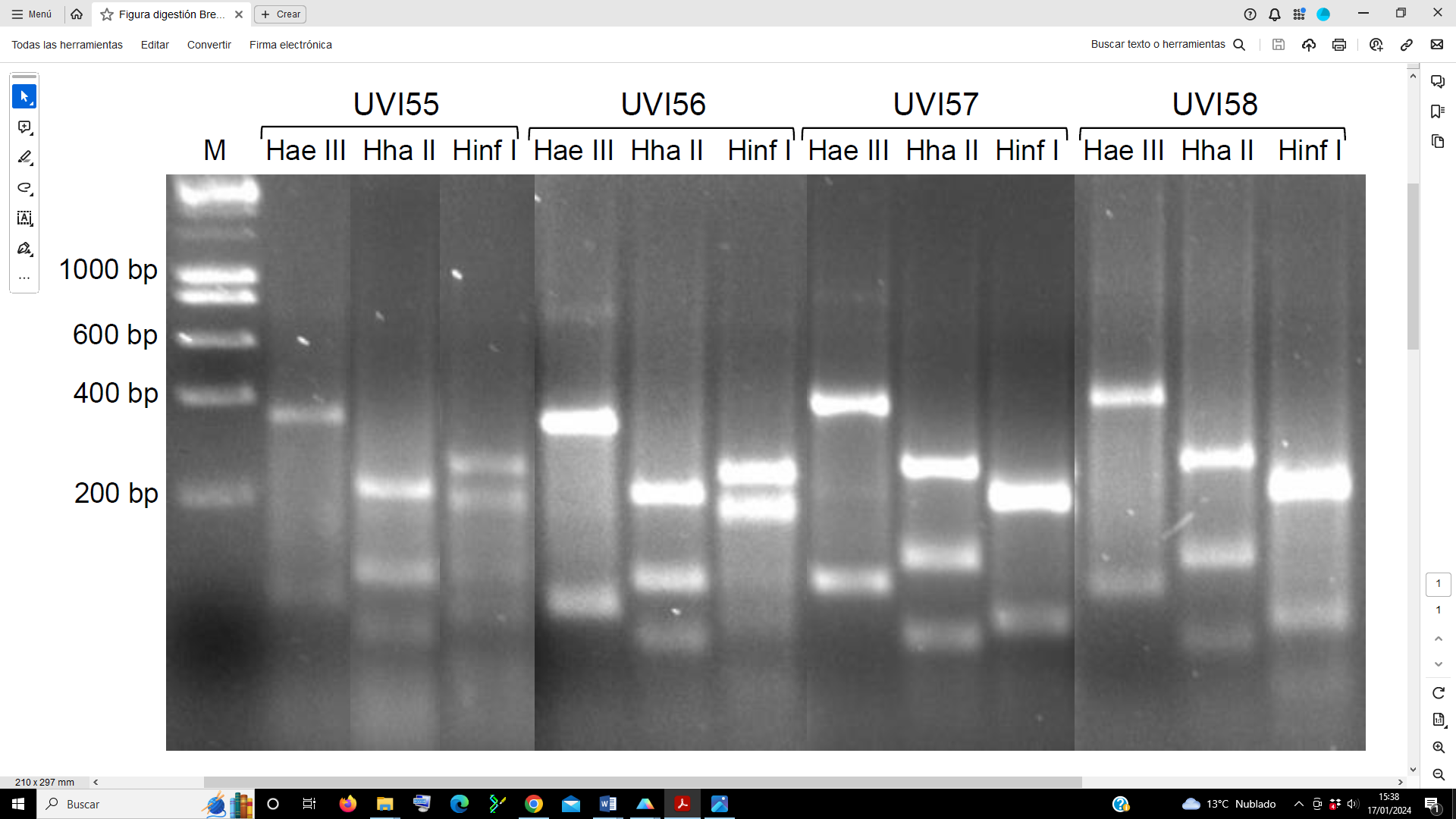


**Figure S2**. Restriction analysis of the ITS/5.85 ribosomal DNA region with the endonucleases *Hae*III, *Hha*II and *Hinf*I. Lane M corresponds to molecular size standard (NZYDNA Ladder III, nzytech).


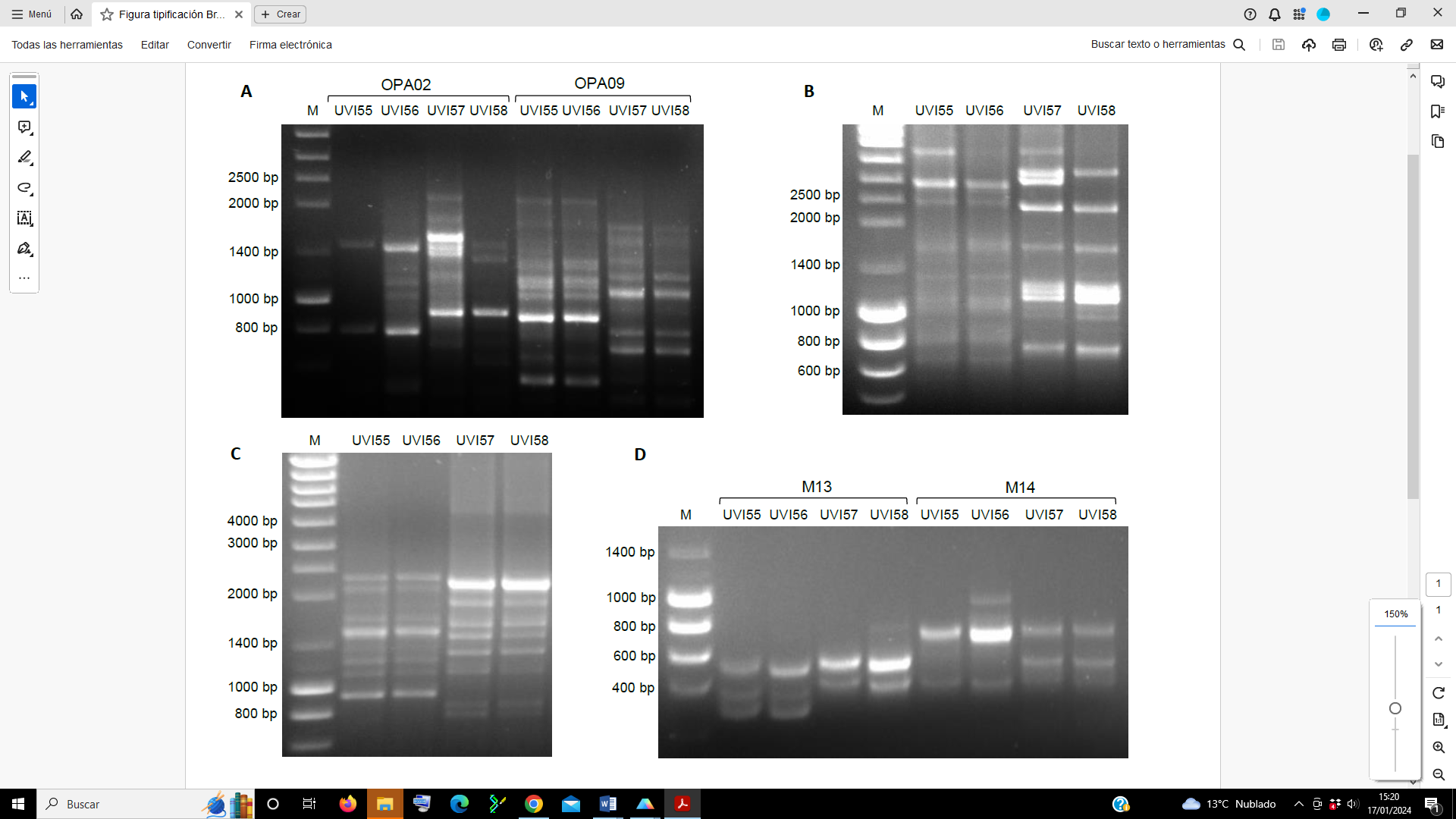


**Figure S3**. Random-amplified polymorphic DNA patterns of the yeast isolates generated with OPA02 and OPA09 (A), GTG_5_ (B), GAC_5_ (C), M13 and M14 (D). Lanes M correspond to molecular size standards (NZYDNA Ladder III, nzytech).

**UVI58**

**UVI57**

**UVI56**

**UVI55**


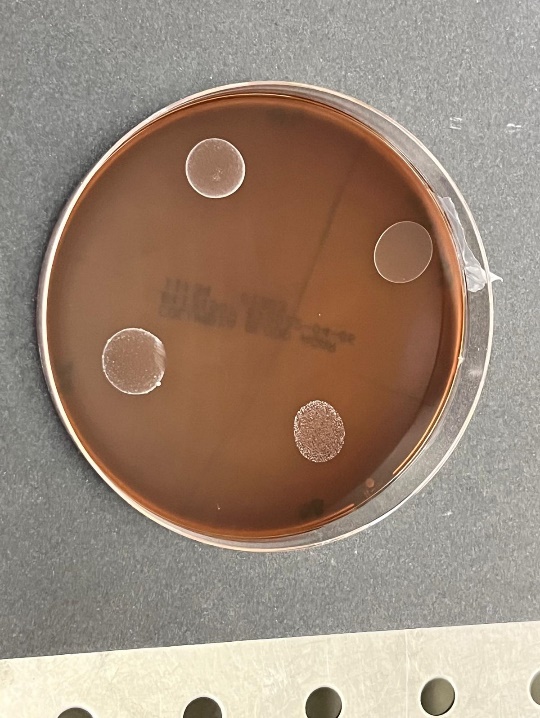


**Figure S4**. Hemolytic activity of yeasts UVI55, UVI56, UVI57 and UVI58.


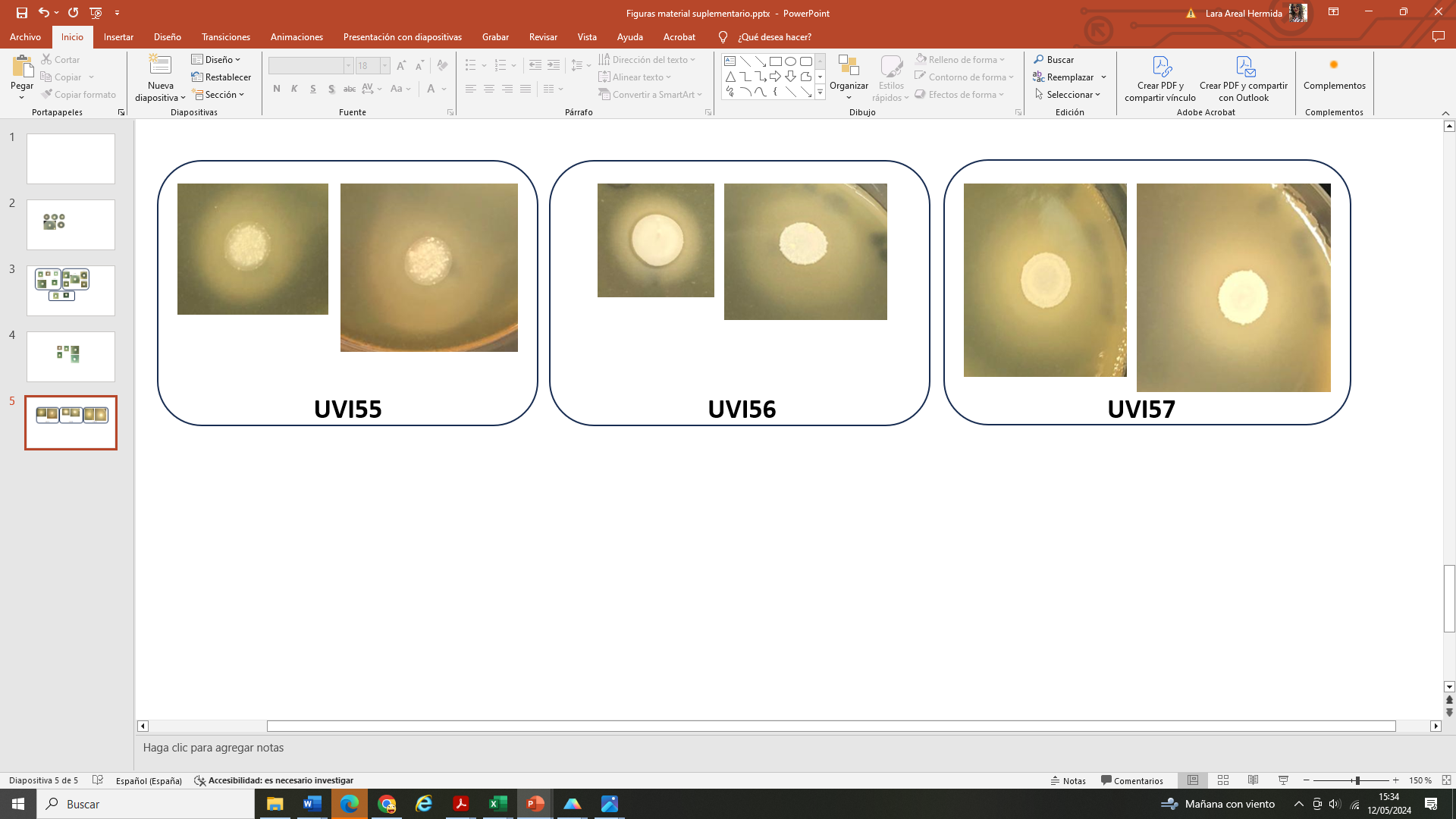


**Figure S5**. Bile Salt hydrolase activity of yeasts UVI55, UVI56 and UVI57 after 48 hours at 30^◦^C (left colonies) and after 6 days at 30^◦^C (right colonies).


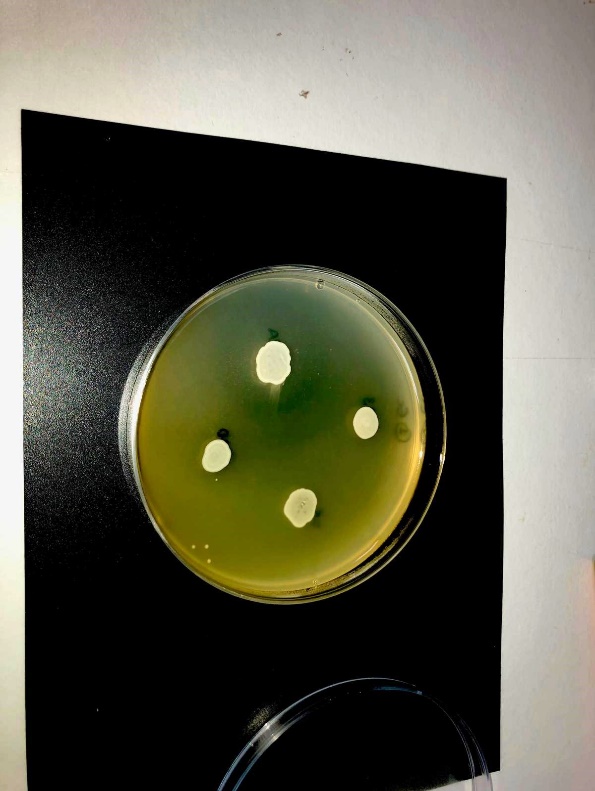


**Figure S6**. Antimicrobial activity of yeasts strains UVI55 (A), UVI56 (B) and UVI57 (C), against different pathogens.


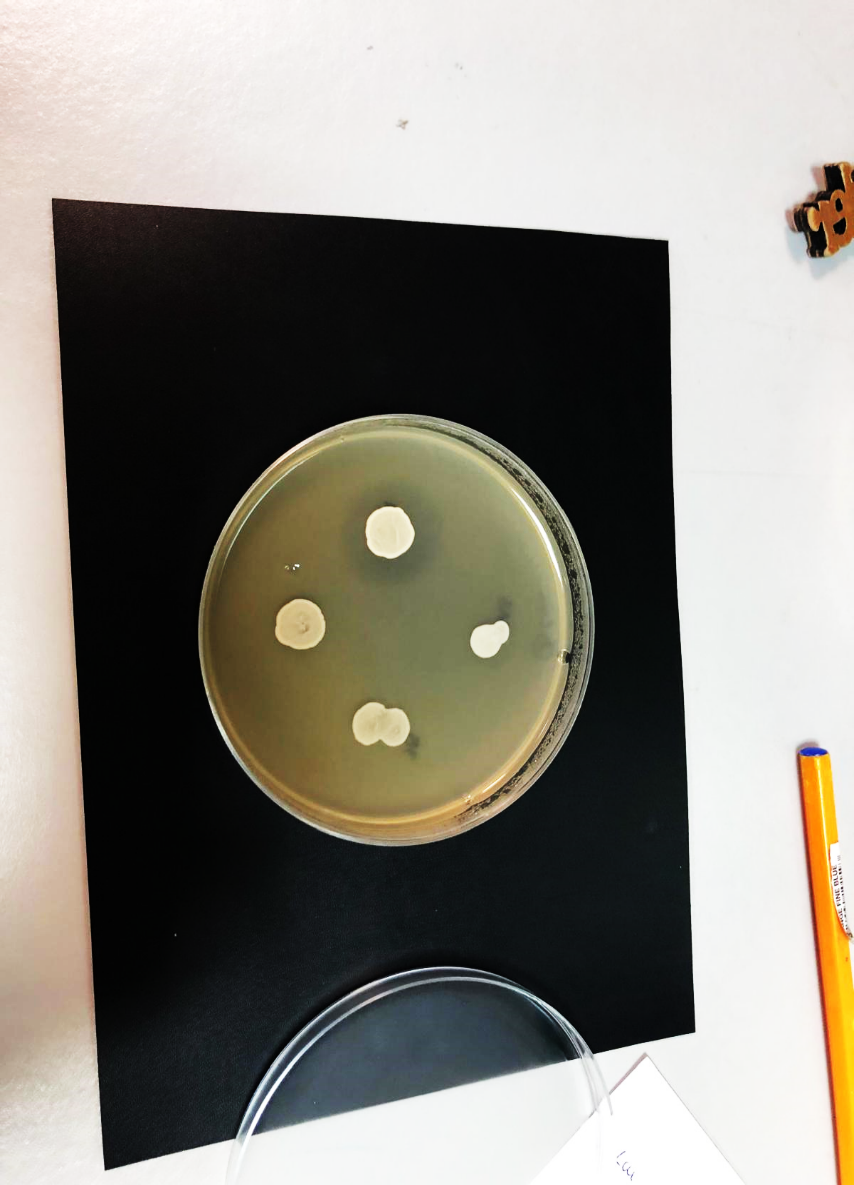


*S. enterica*

*S. aureus*

*E. coli*

*B. cereus*

*L. monocytogenes*

B


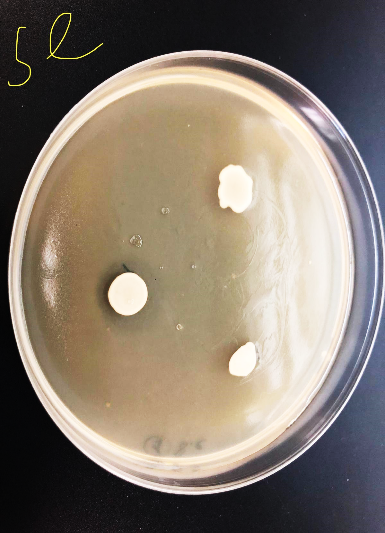

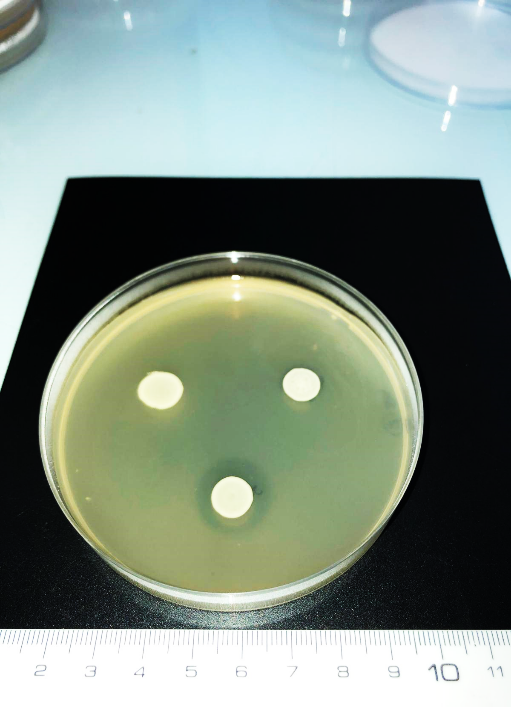

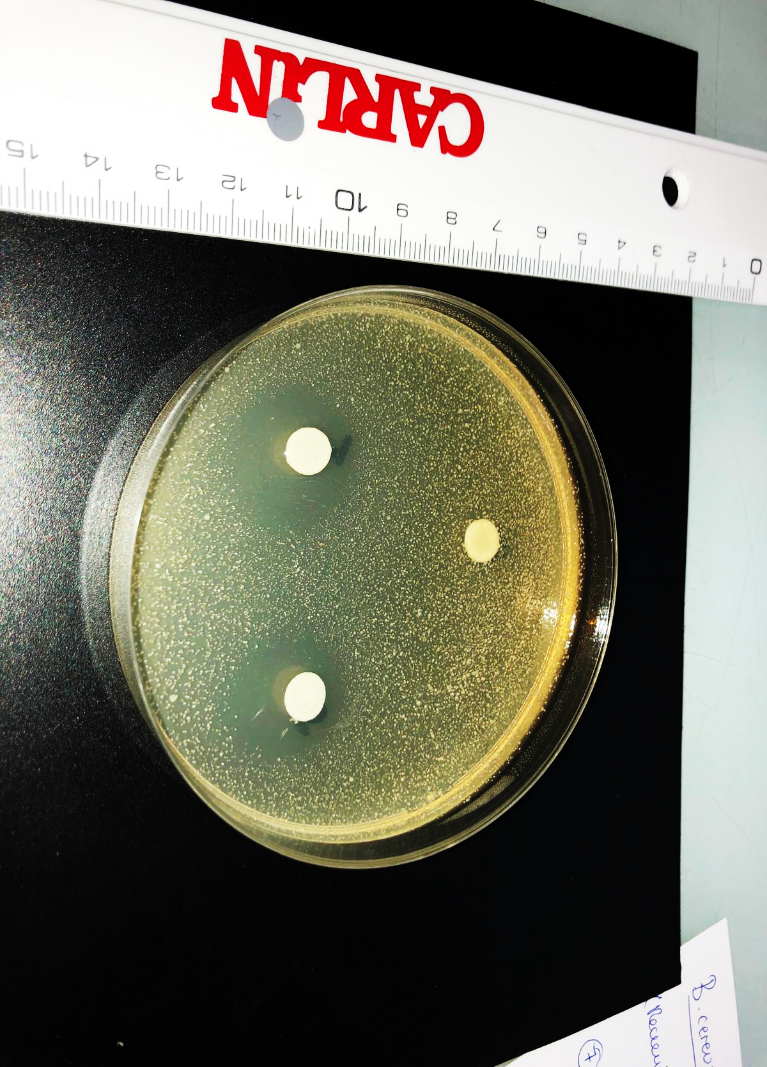

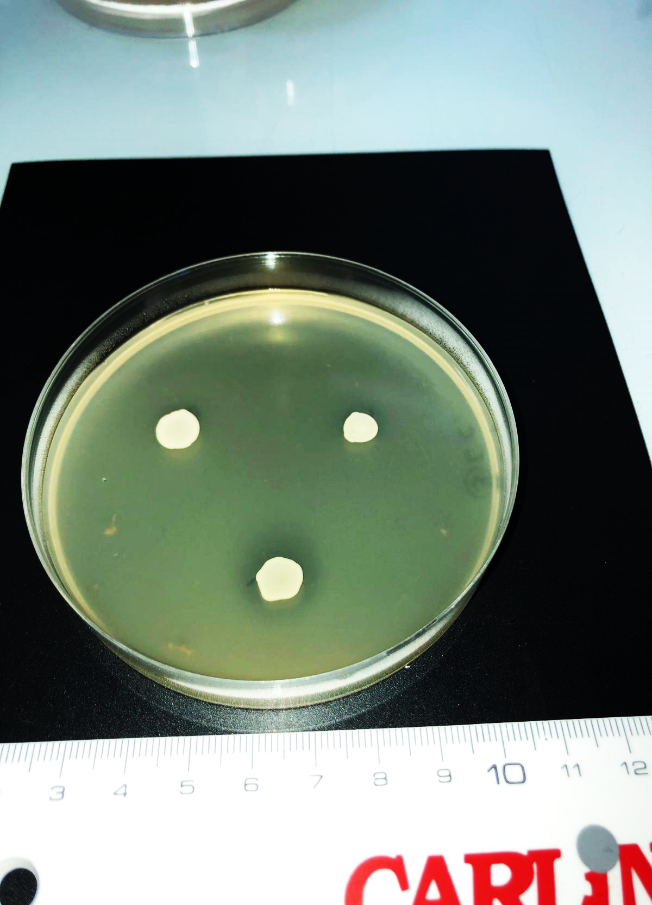


A

*B. cereus*

*S. enterica*

*S. aureus*

*E. coli*

*L. monocytogenes*

*B. cereus*

*L. monocytogenes*

C


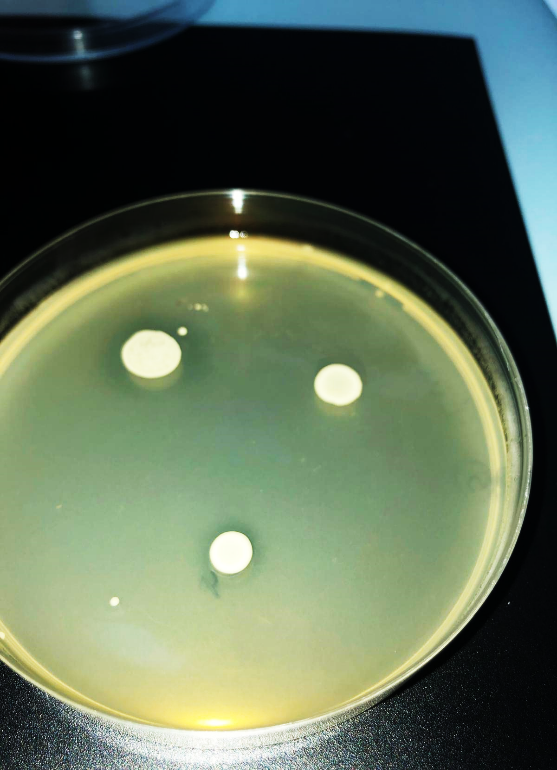

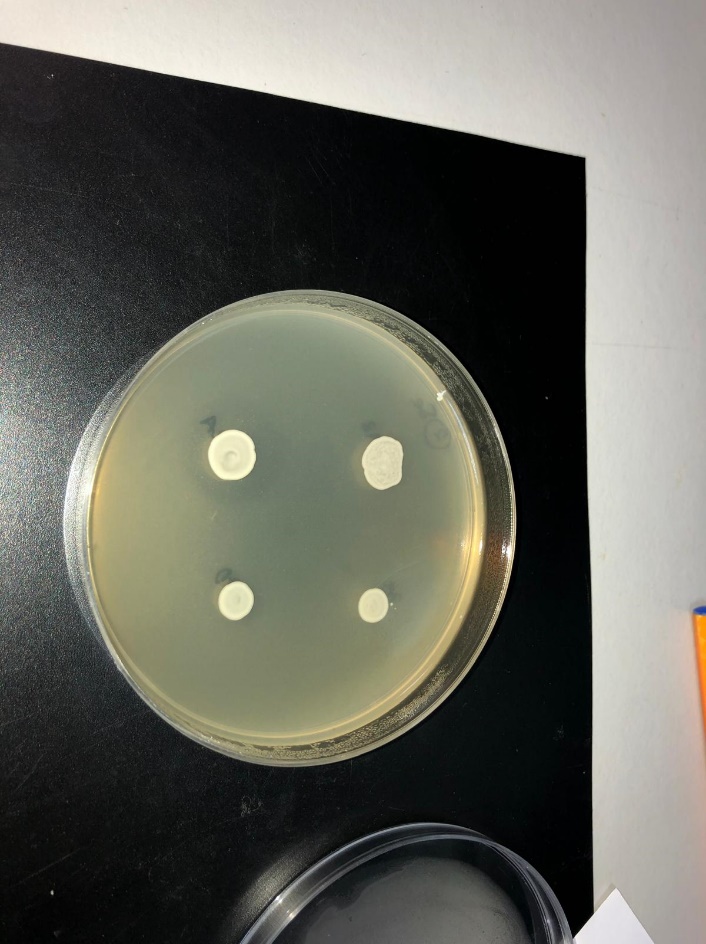

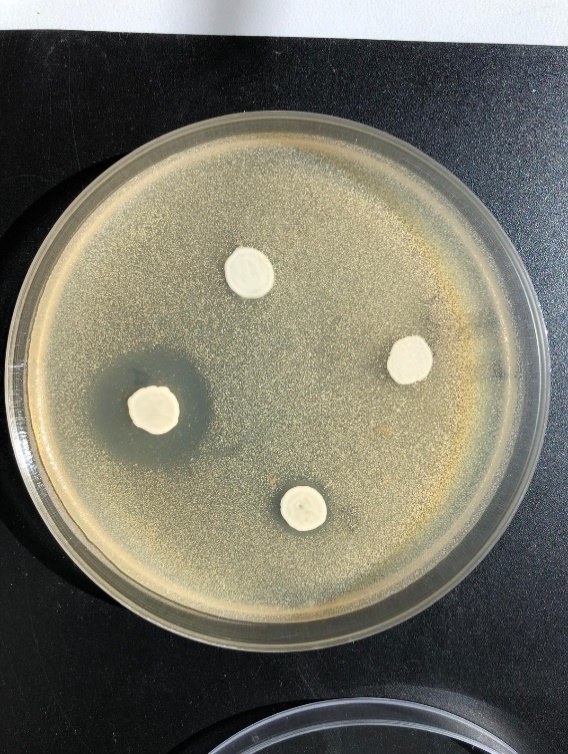

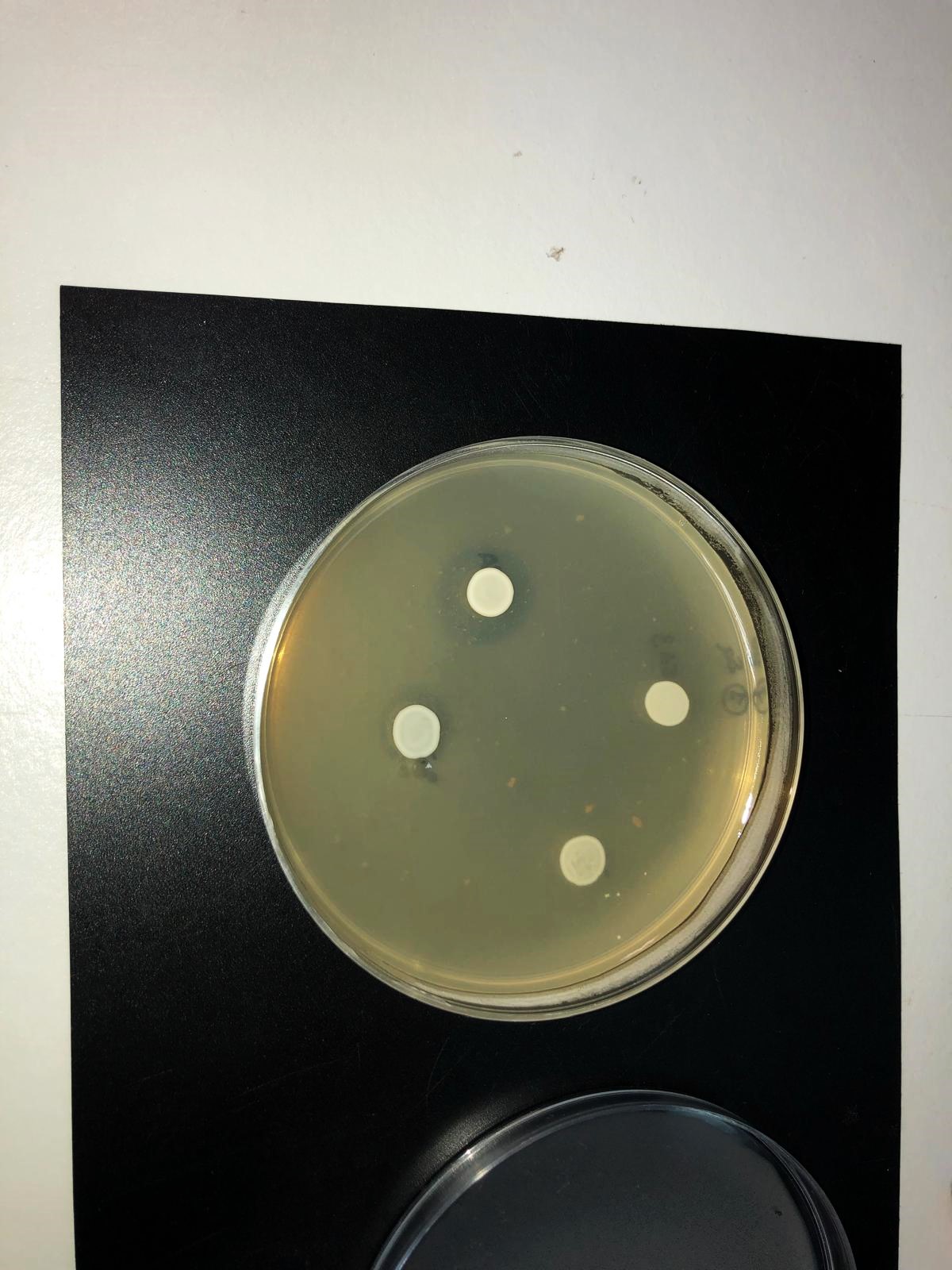

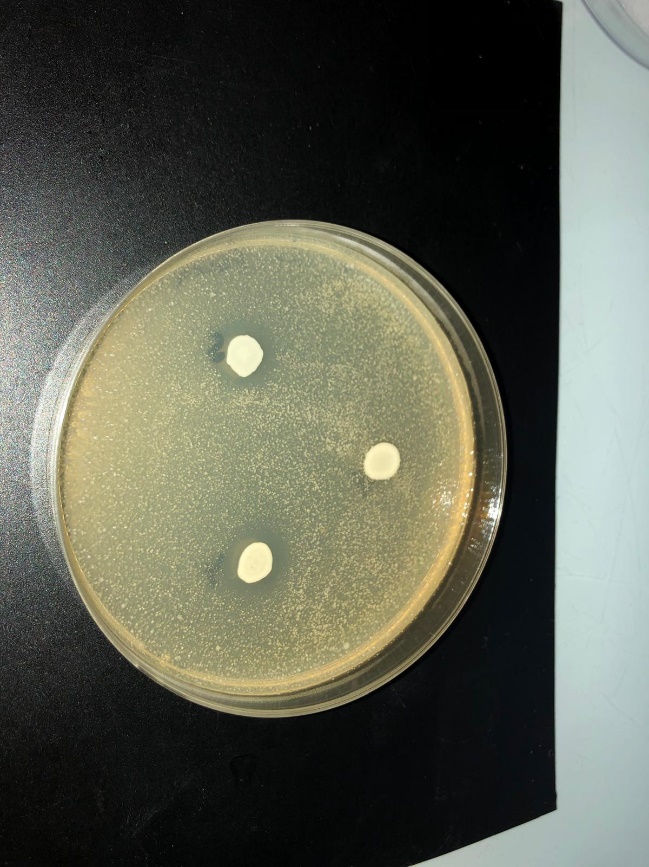

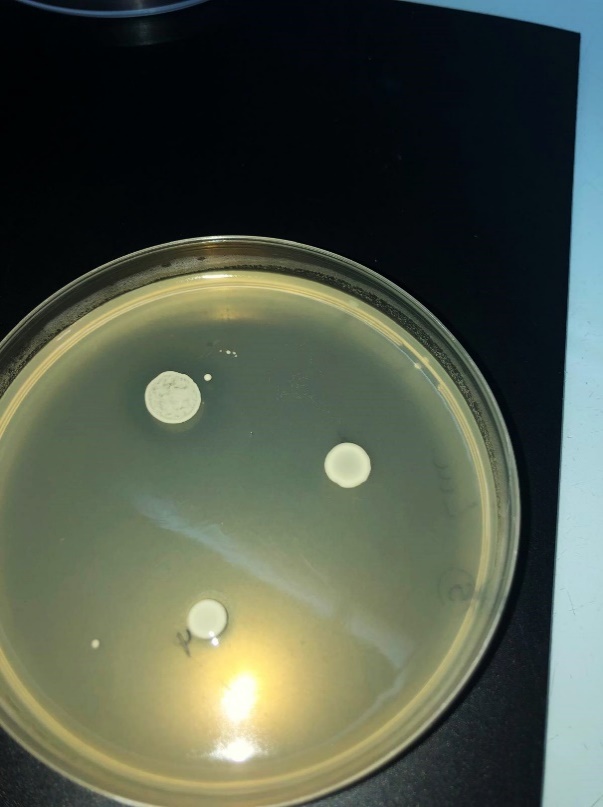


**UVI56**


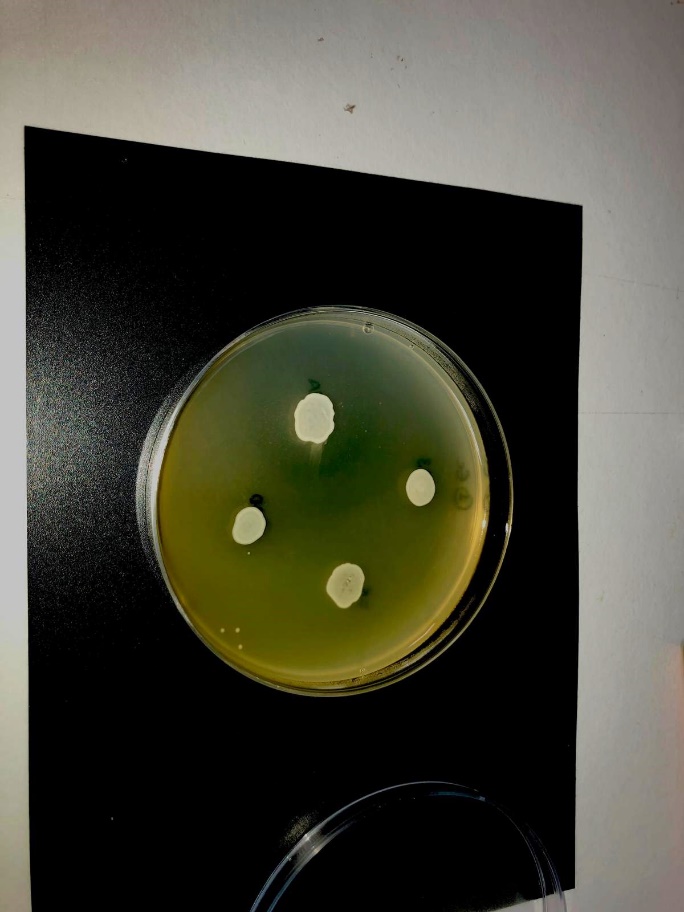

Supplement: Supplementary file 4 [file Table_2.DOCX]
